# Evaluation of a Custom Design Gene Panel as a Diagnostic Tool for Human Non-Syndromic Infertility

**DOI:** 10.3390/genes12030410

**Published:** 2021-03-12

**Authors:** Ozlem Okutman, Julien Tarabeux, Jean Muller, Stéphane Viville

**Affiliations:** 1 IPPTS, Université de Strasbourg, 67000 Strasbourg, France; ookutman@unistra.fr; 2 Laboratoire de Diagnostic Génétique UF de génétique de l’infertilité, Hôpitaux Universitaires de Strasbourg, 67000 Strasbourg, France; 3 Laboratoires de Diagnostic Génétique, UF de Génétique Moléculaire, Hôpitaux Universitaires de Strasbourg, 67000 Strasbourg, France; julien.tarabeux@chru-strasbourg.fr (J.T.); jeanmuller@unistra.fr (J.M.); 4 Laboratoire de Génétique Médicale, INSERM, UMRS_1112, Institut de Génétique Médicale d’Alsace (IGMA), Université de Strasbourg Faculté de Médecine de Strasbourg, 67000 Strasbourg, France; 5 Unité Fonctionnelle de Bioinformatique Médicale Appliquée au Diagnostic (UF7363), Hôpitaux Universitaires de Strasbourg, 67000 Strasbourg, France

**Keywords:** custom design panel, high-throughput sequencing, non-syndromic human infertility, DNA

## Abstract

Infertility is a global healthcare problem, which affects men and women equally. With the advance of genome-wide analysis, an increasing list of human genes involved in infertility is now available. In order to evaluate the diagnostic interest to analyze these genes, we have designed a gene panel allowing the analysis of 51 genes involved in non-syndromic human infertility. In this initial evaluation study, a cohort of 94 non-syndromic infertility cases with a well-defined infertility phenotype was examined. Five patients with previously known mutations were used as positive controls. With a mean coverage of 457×, and 99.8% of target bases successfully sequenced with a depth coverage over 30×, we prove the robustness and the quality of our panel. In total, we identified pathogenic or likely pathogenic variations in eight patients (five male and three female). With a diagnostic yield of 8.5% and the identification of a variety of variants including substitution, insertion, deletion, and copy number variations, our results demonstrate the usefulness of such a strategy, as well as the efficiency and the quality of this diagnostic gene panel.

## 1. Introduction

The demonstration that infertility may in some instances have a genetic basis was provided soon after the first human karyotype was established. Indeed, in the late 1950s, numerical and structural chromosome abnormalities were identified in infertile patients [1]. For many years, karyotyping was the only available test to establish a genetic etiology of infertility. About twenty years later, Tiepolo and Zuffardi demonstrated a correlation between male infertility and Y chromosome deletions [2]. This locus was then defined as the “azoospermia factor” (AZF). However, the complexity of the AZF locus was only uncovered in the mid-1990s when it was subdivided into three sub-genomic regions, AZFa, AZFb, and AZFc, from proximal to distal Yq [3,4]. It was at about the same time that the identification of monogenic causes of male and female infertility began. Since then, the number of monogenic mutations discovered has increased very rapidly, with acceleration in the early 21st century due to the advent of whole-genome analyses, in particular high-throughput sequencing (HTS).

Mutations can be responsible for either syndromic or non-syndromic infertility. Syndromic cases associate infertility with other symptoms, which are usually the patient’s primary concern. With a few exceptions (for example, patients with cystic fibrosis or myotonic dystrophy) these patients, due to their health conditions, don’t have the opportunity to plan a parental project. On the other hand, non-syndromic cases involve patients with infertility not associated with any other symptoms. During the last two decades, large-scale genome-wide analyses of family cases of infertility or groups of infertile patients have identified a fast-growing number of “infertility genes”, solely responsible for non-syndromic infertility. This progress suggests it is now time to provide fertility practitioners with access to a new diagnostic tool for their patients. Unfortunately, only a limited amount of information is available so far from most studies on infertility genes, as nicely underlined by Oud et al. on male infertility [5]. In fact, a majority of the mutations described still await validation. This renders it tricky to choose a panel of genes to include in a diagnostic practice.

Until recently, the genetic diagnosis of infertility was mainly limited to karyotyping, *CFTR* mutation screening, Yq microdeletion testing for azoospermic or severe oligozoospermic patients, and, for women, to *FMR1* gene screening to exclude Fragile X-associated primary ovarian insufficiency (FXPOI). However, the advent of HTS technologies has opened the field. Indeed, gene panel sequencing, allowing the simultaneous analysis of dozens to hundreds of genes, is now the common tool in human genetics, and some laboratories are already dedicating panels to infertility [6,7,8,9,10,11].

We present here the development, validation, and results of our activity based on a panel of 51 genes involved in different forms of non-syndromic male and female infertility.

## 2. Materials and Methods

### 2.1. Studied Population

Male and female patients showing different infertility phenotypes were recruited (Table 1). Germethèque biobank (BB-003-00081), site of Toulouse, (https://www.chu-toulouse.fr/-germetheque-centre-de-ressources-biologiques-, accessed on 3 March 2021), provided 40 male DNA samples extracted from whole blood and their associated data to realize this study [12]. Germethèque obtained consent form from each patient to use their samples (CPP 2.15.27). The Germethèque pilotage committee approved the study design on 1 December 2016. The biobank has a declaration DC-2014-2202 and an authorization AC-2015-2350. The number of the request made to Germethèque is 20161013, and its contract is referenced under the number 17 008 C. For the remaining set, saliva or blood samples were collected from collaborating clinics.

All patients were diagnosed through a complete diagnostic work-up for couple infertility. The minimum requirement was to follow the institutional directives already established for the diagnosis of a pathology being studied.

In the field of male infertility, World Health Organization (WHO) instructions were followed, and at least two detailed spermiograms performed at an interval of at least three months, in order to define the defect in terms of sperm number (azoospermia or oligozoospermia) and the motility of the sperm cells present (asthenozoospermia). If the study was focused on morphological defects (teratozoospermia), both a spermiogram and a spermocytogram were required. The patient may have had a combination of these defects, some of them (oligoasthenozoospermia, asthenoteratozoospermia, oligoteratozoospermia), or all (oligoasthenoteratozoospermia). In the case of non-obstructive azoospermia, it can be worthwhile, but not required, to have information on testis histology. Normal karyotype and absence of Y chromosome microdeletion were required for azoospermic and severe oligozoospermic cases. The workflow described by our group [13] was followed for the diagnosis of male infertility.

For female infertility, there is no defined workflow except for the premature ovarian insufficiency (POI) phenotype; however, a normal karyotype is required in all defined phenotypes. Diagnosis for POI was based on ESHRE recommendations, which defined POI as oligo/amenorrhea for at least 4 months and an elevated FSH level (>25 IU/L) on two occasions > 4 weeks apart (https://www.eshre.eu/Guidelines-and-Legal/Guidelines/Management-of-premature-ovarian-insufficiency.aspx, accessed on 10 February 2021). Additionally, *FMR1* premutation testing was required for POI patients. At least two oocyte pick up (OPU) cycles with identified oocyte maturation defect and estradiol level at the day of OPU were decisive for the diagnosis of women with oocyte maturation defect.

Genomic DNA from patients was extracted from peripheral blood using a QIAamp^®^ DNA Mini kit (Qiagen, Hilden, Germany) or from saliva using an Oragene DNA self-collection kit (DNA Genotek, Ottawa, Canada) according to the manufacturers’ instructions.

A positive control cohort was defined with 5 patients for whom a gene mutation was identified from previous whole-exome sequencing (WES) runs and confirmed by Sanger sequencing. The phenotype of control samples and their previously defined mutations are listed in Table 2.

### 2.2. Gene Panel Design

The gene panel was designed at the beginning of 2017. Gene selection was based on the following criteria:

(i) Infertility genes: Genes defined by Online Mendelian Inheritance in Man (OMIM), at the time of design, as responsible for a non-syndromic male and/or female infertility phenotype; coded as spermatogenic failure (SPGF) for male infertility, and as premature ovarian failure (POF) and as oocyte maturation defect (OOMD) for female infertility.
(ii) Candidate genes: Genes for which at least one variant potentially pathogenic for the related phenotype in humans has been identified by good-quality WES studies, but which need further confirmation.
(iii) *FMR1* sequencing: There is an association between pre-mutation of the *FMR1* gene and increased susceptibility to idiopathic POI. We added *FMR1* on the gene list in order to elucidate possible disease-causing variants for POI.

The Infertility Panel V2, HUS, Strasbourg, France (referred to as “infertility panel” in the rest of text), includes 51 genes in total; comprising 34 genes for male infertility, 15 genes for female infertility, and 2 genes shared by both. Table 3 lists the genes explored in this study; it comprises 33 Infertility genes (IG), 17 candidate genes (CG) and *FMR1* for sequencing (S). In the meantime, among genes listed as “candidate genes”, *NR0B1* (known also as *DAX1*), *Wt1*, and CCDC39 were validated as “infertility genes” with strong or definitive evidence [5].

The Infertility panel included also 6 common single-nucleotide polymorphisms (SNPs). The genotypes of 6 SNPs were used as identity-vigilance markers to verify and monitor the identity of patients by comparing the results obtained using an independent Taqman technology: *PRSS12*, chr4:119,237,348 (rs2292597); *TRAPPC9*, chr8:141,461,062 (rs3735803); *AP4E1*, chr15:51,217,361 (rs2306331); *GRIN2B*, chr12:14,018,777 (rs7301328); *FTCD*, chr21:47,558,473 (rs1047179); *DOCK8*, chr9: 286,593 (rs529208). Control SNPs were selected on the basis of allele frequency (∼0.5) and location on different chromosomes.

The design of the probes was carried out with the Agilent SureSelect application (www.agilent.com/genomics/suredesign, accessed on 4 January 2017). The list of genes and the genomic positions of control SNPs were submitted to SureDesign for the choice of capture probes according to the following specifications: cover the exons of the genes of interest as well as 25 bp of intronic sequence on either side of the exons and without the UTR regions, with 3 probes per nucleotide in the target regions (3× tiling). Coding regions of all the transcripts of the genes of interest were obtained by SureDesign via several sources (Refseq, ensembl, CCDS). The genomic coordinates of the proposed design were assessed in the UCSC genome browser [14] by using genome assembly Human GRCh37/hg19.

### 2.3. Gene Panel Sequencing

The method of detection of constitutional variants by capture and massive parallel sequencing on NextSeq550 has been validated and is used in our medical department (Genetic Diagnosis Laboratory, HUS, Strasbourg, France) in various diagnostic tests [15,16,17].

Libraries were prepared using SureSelectQXT Target Enrichment System (Agilent Technologies, Santa Clara, CA, USA), which uses RNA probes to capture known coding DNA. Briefly, 50 ng genomic DNA was fragmented enzymatically, and specific adaptor oligos were ligated to fragments of the DNA to be sequenced. The probes hybridized on the regions of interest were then captured by a magnetic system (beads coupled to streptavidin). After purification of the enriched DNA fragments, a PCR was carried out in order to increase the number of enriched libraries, which were then double-indexed by oligonucleotide barcodes (a unique barcode per patient within the same series) and pooled by 30 before sequencing on NextSeq 550 System (Illumina, San Diego, CA, USA) with 2 × 75 bp reads for a total 51 genes and 6 control SNPs.

### 2.4. Data Analysis

Sequencing data was analyzed by STARK (Stellar Tools from raw sequencing data Analysis to variant RanKing), our in-house bioinformatics pipeline. STARK adopts the Genome Analysis Toolkit (GATK) recommendations [18]. It performs reads demultiplexing, alignment to the reference human genome (GRCh37/hg19), indel (insertion or deletion) realignment, bam recalibration, variant detection (calling), and variant annotation steps. It takes as input the raw sequencing data (BCL, FASTQ, or unaligned BAM) and generates annotated results (VCF) as well as quality reports. SNV/indels were then annotated and ranked using VaRank [19]. VaRank incorporates the annotations retrieved by the Alamut Batch software (Interactive Biosoftware, France) as well as allele frequency from our internal exome database. Annotations include HGVS nomenclature (genomic, cDNA, and proteic), and population database frequencies from the 1000 Genomes Project ([20], http://www.1000genomes.org/, accessed on 15 February 2021) and gnomAD databases ([21], http://gnomad.broadinstitute.org/, accessed on 15 February 2021). Data processing and analysis were performed as described previously with minor changes [22].

Copy number variants (CNV) have been called using the CANOES program [23] and annotated using AnnotSV [24] with similar databases as SNV/indel, but also including DGV and dbVar.

Variant filtering has been carried out according to allele frequency in 1000 Genomes Project data and in GnomAD (filtering out allele frequency when >1%). Variant classification was performed following guidelines for the interpretation of sequence variants according to the American College of Medical Genetics and Genomics (ACMG)/Association for Molecular Pathology (AMP) [25]. We consider each transmission mode without prioritizing one. Pathogenicity of missense variants were predicted via available software like Provean/SIFT (http://provean.jcvi.org/, accessed on 15 February 2021 [26]) and PolyPhen-2 (http://genetics.bwh.harvard.edu/pph2/, accessed on 15 February 2021 [27]). Adapted ACMG/AMP guidelines for single-gene copy number variants were used for interpretation of CNV [28].

### 2.5. Identity Control and Confirmation of Mutations

The identity of samples was systematically checked via control SNPs by real-time PCR and then compared with the sequencing results. The predesign TaqMan PCR assay was performed independently on the extracted DNA. The amplification was executed on the patient series in simplex with a blank PCR and a control DNA with known genotype according to manufacturer’s protocol (FastStart TaqMan^®^ Probe Master, Merck KGaA, Darmstadt, Germany).

Selected candidate variations have been confirmed via Sanger sequencing. Primers for Sanger have been designed using the Primer3Plus online program for the region harboring the identified variation. Primer pairs were checked via In-SilicoPCR (https://genome.ucsc.edu/cgi-bin/hgPcr, accessed on 8 February 2020) and primer BLAST (https://www.ncbi.nlm.nih.gov/tools/primer-blast/, accessed on 8 February 2020) for the specificity. The region of interest was amplified by using polymerase chain reaction (PCR). Amplification conditions and all primers are listed in Supplementary Table S1. Amplification and size of the PCR product were checked on 2% agarose gel. PCR products were purified, and double-strand sequencing of each DNA fragment was performed by GATC Services, Eurofins Genomics (Ebersberg, Germany). Sequences were aligned on a reference sequence (GrCH37, hg19) by using ApE plasmid editor in order to check variation(s).

## 3. Results

### 3.1. Cohort Description

A total of 94 patients were included in this study, 79 men and 15 women. The phenotype of each individual is presented in Table 1. Male patients were classified into four categories: sperm production defect (SPD) (50 cases), either azoospermia or oligozoospermia; teratozoospermia (7 cases); asthenozoospermia (1 case); and a mixed phenotype (21 cases). Female patients were classified into two categories, including oocyte maturation defect (OOMD; 4 cases) and premature ovarian insufficiency (POI; 11 cases).

### 3.2. Validation of the Infertility Panel and Identity Control

Among the five patients we used as controls, there were 2 non-obstructive azoospermia patients; C1 had a hemizygous stop-loss in *MAGEB4* [29] and C2 had a homozygous stop-gain in *TEX15* [22]. The remaining three samples were from globozoospermia patients, C3, C4, and C5, who carried either deletions and/or point mutations in *DPY19L2* [30]. All control sample variants could be redetected by the panel analysis (Table 2, Supplementary Figures S1–S4).

The identity of all samples was checked via the included SNPs by TaqMan amplification. All results matched with sequencing results and confirmed the identity of the samples.

### 3.3. Sequencing Results and Identification of Variants

The total panel size was 187 kb with 57 loci comprising 883 distinct regions. Overall, the mean coverage was 457×, and 99.8% of target bases were successfully sequenced with a minimum depth of coverage of 30× (see Supplementary Tables S2 and S3, per gene and per patient, respectively). Genes were analyzed according to the patient’s phenotype. Targeted sequencing and variant analysis statistics are given in Supplementary Table S3. After filtering for allele frequency in the general population (filtered out >1%), predictions of the effect on protein function and a literature check were conducted. We retained causative mutations explaining the infertility phenotype for eight patients (8/94; 8.5%); five male patients (5/79; 6.3%) and three female patients (3/15; 20%). No pathogenic CNV was found related to studied phenotype. Detailed information of the mutations identified and relevant patient information are shown in Table 4.

**Figure 1:**
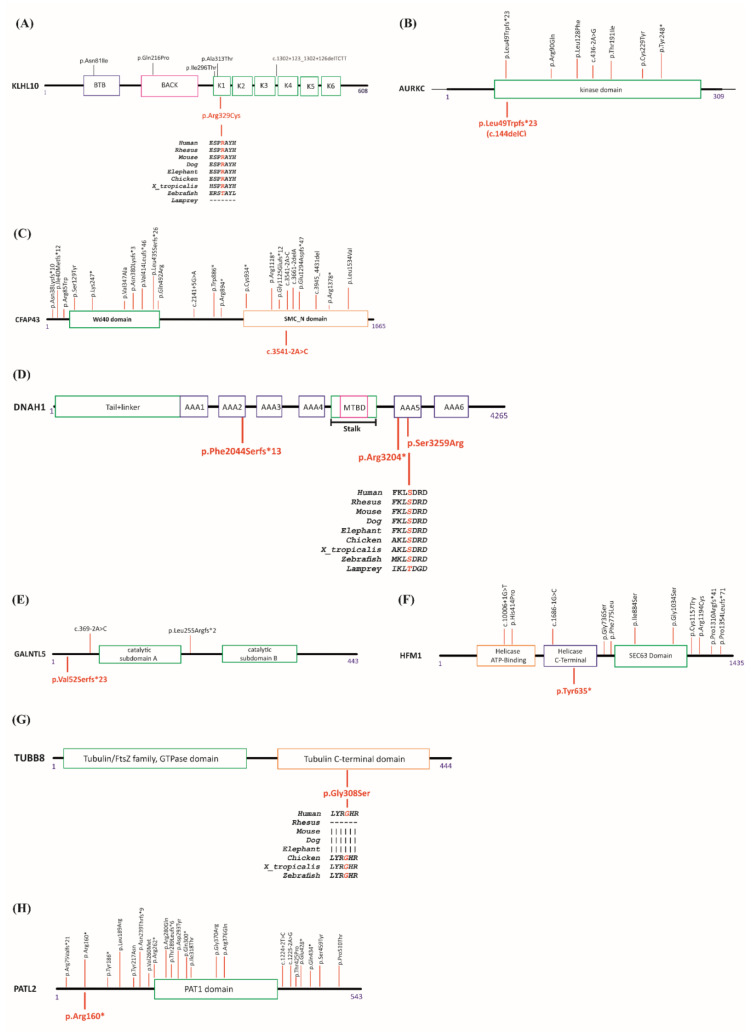
Schematic representation of (**A**) KLHL10, (**B**) AURKC, (**C**) CFAP43, (**D**) DNAH1, (**E**) GALNTL5, (**F**) HFM1, (**G**) TUBB8, and (**H**) PATL2 proteins and their published mutations. Published mutations are indicated on top, while mutations identified in this study are shown at the bottom. Since more than 40 mutations have been identified for TUBB8 and DNAH1, identified mutations are not shown on the figure. Details of mutations on relative genes are given in Supplementary Table S4. Protein sequence alignments for different species are shown for missense mutations identified in this study.

Among the identified mutations, three of them, affecting *PATL2*, *AURKC*, and *CFAP43*, were already described in at least one patient with a related phenotype. In addition, we identified five new mutations affecting *HFM1*, *GALNTL5, KLHL10*, *DNAH1*, and *TUBB8*. Schematic diagrams of the relevant genes and the known mutations are shown in Figure 1A–H and Supplementary Table S4.

Among male patients, patient Pt12 was diagnosed with azoospermia. We identified a heterozygous missense mutation (c.985C > T, p.Arg329Cys) in *KLHL10*. The encoded protein is involved in the ubiquitination process and subsequent proteasomal degradation of target proteins during spermatogenesis. The variation is in the conserved Kelch1 domain (Figure 1A), as is the missense mutation previously identified in one azoospermic patient [31]. Prediction tools indicate a possible damaging/deleterious effect (PolyPhen-2 and Provean/SIFT, respectively).

Patient Pt41 was a 30-year-old man of Algerian descent; his spermiogram showed oligo-astheno-teratozoospermia with total head abnormality. Panel analysis revealed the recurrent homozygous mutation (c.144delC, p.Leu49Trpfs*23) in *AURKC*. The gene product plays a role in meiosis and more particularly in spermatogenesis. With a lack of functional AURKC protein, chromosomal segregation would be perturbed. The identified mutation has been described as the most frequent one in the Maghrebian population [32]. A list of known *AURKC* mutations is given in Figure 1B.

Patient Pt55 was a 28-year-old Moroccan man suffering infertility due to a sperm flagellar problem. Analysis revealed a homozygous splice mutation (c.3541-2A > C, p.?) in *CFAP43*. This gene encodes a protein involved in sperm flagellum axoneme organization and function. The same mutation was reported before in two unrelated Tunisian patients homozygously for sperm flagella problems [33]. A list of known CFAP43 mutations is given in Figure 1C.

Patient Pt65 was a 33-year-old male of French origin, suffering from severe astheno-teratozoospermia, with 97% immotile sperm and 92% abnormal flagella. Three heterozygous variations in *DNAH1* were identified, one frameshift (c.6131del, p.Phe2044Serfs*13) giving rise to a premature stop codon thirteen codons downstream from the start codon, a stop gain mutation (c.9610C > T, p.Arg3204*), and one missense (c.9777T > G, p.Ser3259Arg). The missense variation, which is located in a conserved region, is predicted as deleterious or possibly damaging using the prediction tools Provean/SIFT and PolyPhen-2 respectively (Figure 1D). *DNAH1* gene product is required in spermatozoa for the formation of the inner dynein arms and biogenesis of the axoneme; it is an energy-generating protein needed for sperm motility. In order to establish the allelic distribution of these variants, we analyzed his parents by Sanger sequencing. He inherited two variations from his mother (c.6131del, p.Phe2044Serfs*13 and c.9777T > G, p.Ser3259Arg) and one heterozygous variation from his father (c.9610C > T, p.Arg3204*), ruling out the possible involvement of the missense in the patient’s phenotype. His brother was also tested; he carried the same mutations and suffered from the same infertility problems.

Patient Pt77 was a 29-year-old male; his spermiogram was diagnosed as oligo-astheno-teratozoospermia with 90% immotile sperm. Our analysis revealed a heterozygous frameshift mutation (c.153dup, p.Val52Serfs*23) in *GALNTL5* (Figure 1E). The functional gene product is essential for mammalian sperm formation. The identified mutation theoretically leads to an early translational termination at the very beginning part of the protein.

Among female patients, patient Pt2 was a 30-year-old woman of Turkish descent. She had low anti-Müllerian hormone (AMH) (0.04 ng/mL) and a normal karyotype, and her parents were first degree cousins. The patient had normal *FMR1* alleles with 23 and 30 CGG repeats. Our panel gene analysis revealed a homozygous stop gain mutation (c.1905T > A, p.Tyr635*) in *HFM1*. The list of known mutations is given in Figure 1F.

Patient Pt 38 was a 32-year-old woman from a Turkish consanguineous family. She had a history of 12 years of primary infertility. She underwent four failed in vitro fertilization (IVF) attempts; all retrieved oocytes were either degenerate or immature. We identified a homozygous missense mutation (c.922G > A p.Gly308Ser) in *TUBB8*. The mutation was located in the conserved C-terminal domain of the protein and was predicted as possibly damaging or deleterious by PolyPhen-2 and Provean/SIFT respectively (Figure 1G).

Patient Pt 71 was a 31-year-old woman of Tunisian descent with primary infertility and a history of consanguinity in her family line. She had undergone three failed IVF cycles. Seven to 30 oocytes were retrieved in each cycle; however, all of the oocytes were at a germinal vesicle stage or atretic. She carried a homozygous stop gain mutation, (c.478C > T, p.Arg160*) in *PATL2*. This mutation was previously listed as a causative mutation in patients with oocyte maturation arrest [34]. The list of previously known *PATL2* mutations is given in Figure 1H.

## 4. Discussion

The recent improvement of whole genome analysis has allowed an exponential rate of infertility gene identification. Indeed, during the last two decades, the number of genes proven to be involved in male or female non-syndromic infertility has increased very quickly. As for many, if not all medical fields, the time has come to translate this research to the clinical setting.

The goal of the present study was to assess the clinical value of a diagnostic gene panel in the ART practice and to establish the prevalence of mutations in selected genes for the cohort of patients studied. Diagnostic yields of available infertility panels worldwide range between 0.4% to 25% (Table 5). It is worth noting that results provided by Canerella et al. are confusing, and their diagnostic yield of 25% seems to be overestimated by collapsing results with their previous study [10]. However, available reports are difficult to compare because the list of genes, the studied phenotypes, the number of patients analyzed, and the quality of HTS are different in each study. Most of them do not report the quality of their sequencing, or do so only partially, which should be: (1) high sequence coverage of 100× to guarantee sequence of whole gene, which is compensatory for the detection of all rare variants; (2) more than 95% of the region of interest covered by at least 30 reads for a good quality HTS study [35]. In addition, most of these studies do not provide patients’ clinical data, and none of them analyze CNVs. It is almost certain that the diagnostic yield will be improved nonetheless by the HTS quality, but also by the strict gene selection and the number of genes analyzed.

For this purpose, we set up a panel of 51 genes responsible for a non-syndromic infertility phenotype. Our control cohort was chosen in order to challenge as much as possible the ability of our panel to detect various types of variants. Indeed, we show that our strategy allows us to identify substitutions, indels, and CNVs. Subsequently, we analyzed 94 patients (79 men and 15 women) with precise infertility phenotypes. We have shown the efficiency and the quality of our panel with a mean coverage of 457× and 99.8% of target bases successfully sequenced with a depth coverage over 30×. In total, we identified causative mutations for eight of the tested patients (8.5%; 8/94), five for the male cohort (6.3%; 5/79) and three for the female patients (20%; 3/15). Mutations identified in three patients in the present study have already been reported and new mutations were identified in five patients.

Among male patients, a new heterozygous missense mutation in *KLHL10* was identified in an azoospermic patient. OMIM gives an autosomal dominant inheritance for *KLHL10* mutations. Initially, *KLHL10* gene mutations were mainly identified in oligospermic patients [36]; however, a recent study described a mutation in *KLH10* in an azoospermic patient [31]. Therefore, we cannot rule out that mutations in this gene may lead to an oligozoospermia evolving towards a complete azoospermia over time. Unfortunately, we have only recent spermiograms for this patient. Further investigations are critical to confirm this finding. Indeed, if mutations in *KLHL10* imply an evolution from oligozoospermia to azoospermia, this could impact the care proposed to such patients and their relatives. It is important to offer patients, as soon as possible, sperm cryopreservation, and also important to test brothers and other male relatives in order to propose cryopreservation for the carriers.

A second patient was suffering from macrozoospermia, and he carried the recurrent *AURKC* mutation found in the North African population. Overall, with full *AURKC* gene sequencing, a positive mutation diagnosis is found in 83.7% of macrozoospermic patients [37]. Although different homozygous or heterozygous mutations have been identified, two mutations in *AURKC* are recurrent. The first one, c.144delC (p.Leu49Trpfs*23), is found in North African populations, and the other, c.744C  >  G (p.Y248*), is found in European populations. For the first one, it has been clearly demonstrated that in such a situation, the majority of spermatozoa are tetraploid, therefore the only ART option possible is sperm donation [38]. OMIM defines the pathology linked to *AURKC* as transmitted under an autosomal recessive mode.

For a third patient showing MMAF, we found a previously reported homozygote mutation in *CFAP43*. Autosomal recessive inheritance was reported in OMIM for *CFAP43* mutations in men with MMAF, including absent, short, coiled, bent, and irregular-caliber flagella (Figure 1C). It seems that ICSI can be safely proposed, with a reasonable success, to these patients [39]. Next, we identified in a patient diagnosed with severe astheno-teratozoospermia three heterozygous variations in *DNAH1*, two transmitted by his mother and one by his father. So far, more than forty mutations have been identified in *DNAH1* as possible causes of MMAF (Figure 1D and Supplementary Table S4. WES was the common technique in all published studies, and, except for two, all patients were homozygous or carried two heterozygous mutations. This supports a recessive mode of transmission as reported by OMIM. Here again ICSI can be safely proposed, with a reasonable success.

The last male patient, showing an oligo-astheno-teratozoospermia, had a new heterozygous frameshift variation, possibly disease-causing, in *GALNTL5*. Human *GALNTL5* consists of nine exons and codes for 443 amino acid (aa) protein. The variation we identified is on exon 2 (aa52); it is a nucleotide duplication causing a frame shift, creating a premature stop codon and leading to an early translational termination of 23aa. A different heterozygous one-nucleotide deletion has been identified in exon 6 of *GALNTL5* as being causative for male fertility due to immotile sperm [40]. These results have been confirmed in mice. Indeed, heterozygous mutations affected male mice fertility due to immotile sperm [40]. This strongly supports a dominant mode of transmission, although it is not listed in OMIM. *GALNTL5* was classified as CG, though our results confirm and re-inforce genotype/phenotype relation in the asthenozoospermia phenotype in men, therefore contributing to the upgrade of *GALNTL5* as IG.

Among the female patients, one presented a POI, and she carried a new homozygote mutation in *HFM1*. Initially, compound heterozygous mutations in *HFM1* were identified in women with POI [41], and the inheritance mode was given as autosomal recessive by OMIM. Subsequently, it was postulated that heterozygous missense mutations might also be associated with POI [42,43]; however, this needs to be further investigated, since in the first study, using WES, the mother was a heterozygous carrier, and she was reported as clinically normal [41].

The second mutated woman produced, following ovarian stimulation, only degenerate or immature oocytes. She carries a new homozygous mutation in *TUBB8*. As of January 2021, 98 different heterozygous or homozygous mutations in *TUBB8* have been identified as a cause for oocyte maturation arrest in females, and the list is still growing (Supplementary Table S4. Both an autosomal dominant and autosomal recessive mode of inheritance are indicated in OMIM.

The third woman diagnosed by our panel showed an oocyte maturation arrest at the germinal vesicle stage, with an already identified homozygous mutation in *PATL2*. Patients with mutations in *PATL2* can present variable phenotypes, with some oocytes exhibiting maturation arrest at the germinal vesicle stage and others at the metaphase I stage, as well as fertilization failure or, in those that are fertilized, early embryonic arrest. So far, about twenty homozygous or compound heterozygous *PATL2* mutations have been identified in women with oocyte maturation arrest, confirming a recessive mode of transmission as given in OMIM.

The diagnostic yield of our custom designed panel in the present study was 8.5%. Such a result is one of the highest reported so far in this field, but remains lower than for other medical specialties. For instance, a success rate of 25% was reported for the diagnosis of intellectual disability using targeted HTS [15]. These results can be, most probably, explained by the heterogeneity of infertility. This success rate will certainly be improved by including the latest genes identified, increasing the cohort of patients, and narrowing the inclusion criteria of infertility phenotypes.

We are at a transition period where basic research is translated into clinical practice. This will have many positive consequences for patients as well as for ART practitioners. First of all, for an increasing number, it will pinpoint the etiology of the infertility, which is a relief both for patients and their doctors. Precise diagnosis also opens the way to offer genetic counseling for patients, as well as their relatives. Moreover, targeted sequencing studies are valuable to re-classify reported CG genes related to infertility as IG based on the identification of new patients showing the same phenotype and sharing mutations in the same gene.

Having a diagnosis will improve patient care by adapting treatment to the patient’s situation. Being at the beginning of this genetic activity, there are, so far, only a few genes for which a specific action can be proposed. This is the case for *DPY19L2*, where artificial oocyte activation must be offered [44]; *AURKC*, for which sperm donation or renouncing parenthood are the only possibilities [32]; and *TEX15*, where sperm cryopreservation has to be proposed to the patient, but also to his affected brothers even if they do not yet have a parental project [22]. Similar to *TEX15*, *KLHL10* mutations may correlate with a decrease in sperm count over time, and sperm cryopreservation might be proposed to the patient. Considering the low cost of the technique, it could be proposed even before acquiring proof that the sperm concentration will decrease with time.

In order to improve patient care through the genetic diagnosis of infertility, more studies have to be carried out. We are here at the frontier of two activities; research will enrich the diagnostic tools offered, and the diagnostic practice will allow a better definition of the genotype/phenotype correlations to enable personalized care. Indeed, the in-depth analysis of clinical data will allow us to better define the criteria for each genetic test and therefore improve the efficiency of the proposed diagnoses.

## 5. Conclusions

Our custom designed infertility panel is validated and proved to be able to detect various types of variants including substitutions, indels, and CNVs. In total, we identified causative mutations for eight of the tested patients (8.5%; 8/94). The quickest improvement of diagnosis based on panel analysis will come from increasing the number of genes analyzed.

## Figures and Tables

**Table 1:** Infertility phenotype and sex of the recruited patients. The number of patients in each group is indicated.

Sex	Infertility Phenotype		#Patients
Male	Teratozoopsermia		7
	Asthenozoospermia		1
	Sperm production defect (SPD)	Azoospermia	30
		Oligozoospermia	20
	Mixed phenotype		21
Female	Oocyte maturation defect (OOMD)		4
	Premature ovarian insufficiency (POI)		11

#Patients: Number of patients.

**Table 2:** Control samples: phenotype, zygosity, and mutation information. SPD: Sperm production defect, hom: homozygous, het: heterozygous, hemi: hemizygous.

Sample	Phenotype	Mutated Gene	Zygosity	Defined Mutation
C1	SPD	*MAGEB4*	hemi	p.*347Cysext*24 (c.1041A > T)
C2	SPD	*TEX15*	hom	p.Tyr710* (c.2130T > G)
C3	Teratozoospermia	*DPY19L2*	Het gene del and point mutation	Heterozygous *DPY19L2* deletion with p.Arg290His (c.869G > A)
C4	Teratozoospermia	*DPY19L2*	Hom	exon 5-exon 6 deletion in *DPY19L2*
C5	Teratozoospermia	*DPY19L2*	Hom	del *DPY19L2*

**Table 3:** Selected genes for infertility panel: 34 genes related to non-syndromic male infertility, 15 genes related to female infertility, [13 genes for premature ovarian insufficiency (POI), and 2 genes for oocyte maturation defect (OOMD)]; 2 shared genes for non-syndromic male and female infertility have been included in the panel.

**MALE INFERTILITY**	**Phenotype**	**Gene Name**	**OMIM #**	**RefSeq**	**IG/CG**
	Teratozoospermia	AKAP4	300185	NM_003886.2	CG
		AURKC	603495	NM_001015878.1	IG
		BRDT	602144	NM_001242806.2	IG
		CFAP43	617558	NM_025145.6	IG
		CFAP44	617559	NM_001164496.1	IG
		DNAH1	603332	NM_015512.4	IG
		DPY19L2	613893	NM_173812.4	IG
		MTUS1 **	609589	NM_001001924.2	CG
		PICK1	605926	NM_001039583.1	CG
		SEPT12	611562	NM_144605.4	IG
		SPATA16	609856	NM_031955.5	IG
	Asthenozoospermia	CATSPER1	606389	NM_053054.3	IG
		GALNTL5	615133	NM_145292.3	CG
		SLC26A8	608480	NM_001193476.1	IG
		SPAG17	616554	NM_206996.3	CG
	Sperm production defect (SPD)	CCDC39	613798	NM_181426.1	CG *
		DNAH6	603336	NM_001370.1	CG
		HIWI (PIWIL1)	605571	NM_004764.4	CG
		HSF2	140581	NM_004506.3	CG
		KLHL10	608778	NM_152467.4	IG
		MAGEB4	300153	NM_002367.3	CG
		MEIOB	617670	NM_001163560.2	IG
		NANOS1	608226	NM_199461.3	IG
		NPAS2	603347	NM_002518.3	IG
		NROB1 (DAX1)	300473	NM_000475.4	CG *
		SOHLH1	610224	NM_001012415.2	IG
		SPINK2	605753	NM_001271722.1	IG
		TAF4B	601689	NM_001293725.1	IG
		TEX11	300311	NM_001003811.1	IG
		TEX14	605792	NM_001201457.1	IG
		TEX15	605795	NM_031271.3	IG
		Wt1	607102	NM_024426.3	CG *
		ZMYND15	614312	NM_001267822.1	IG
	Total fertilization problem	PLCZ1	608075	NM_033123.3	IG
	**Phenotype**	**Gene Name**	**OMIM**	**RefSeq**	**IG/CG**
**FEMALE INFERTILITY**	Primary Ovarian Insufficiency (POI)	BMP15	300247	NM_005448.2	IG
		FIGLA	608697	NM_001004311.3	IG
		FMR1	309550	NM_002024.5	S
		FSHR	136435	NM_000145.3	CG
		GDF9	601918	NM_005260.5	IG
		HFM1	615684	NM_001017975.4	IG
		MCM8	608187	NM_001281521.1	IG
		MCM9	610098	NM_017696.2	CG
		MSH4	602105	NM_002440.3	CG
		NANOS3	608229	NM_001098622.2	CG
		NOBOX	610934	NM_001080413.3	IG
		PGRMC1	300435	NM_006667.4	CG
		STAG3	608489	NM_001282717.1	IG
	Oocyte Maturation Defect (OOMD)	TUBB8	616768	NM_177987.2	IG
		PATL2	614661	NM_001145112.1	IG
		**Gene Name**	**OMIM**	**RefSeq**	**IG/CG**
Male/Female infertility (SPD/POI)		NR5A1	184757	NM_004959.4	IG
		SYCE1	611486	NM_001143764.1	IG

IG: infertility genes, CG: candidate genes, S: gene to sequence, (*): Candidate genes that were validated since the beginning of this study as strongly or definitely linked to male infertility, (**): genes identified through in-house screening project, not published.OMIM #: OMIM number.

**Table 4:** Identified mutations according to phenotype and sex of the patients.

Sex	Patient Code	Phenotype	Gene Name (Refseq Id)	Coding Effect	Zygosity	Consanguinity	cNomen	pNomen	Allele Frequency (gnomAD)	ART Option
M	Pt12	SPD (Azoospermia)	*KLHL10* (NM_001329595.1)	Missense	Het	NP	c.985C > T	p.Arg329Cys	0.00001202	Cryo- preservation *
	Pt41	Teratozoopermia	*AURKC* (NM_001015878)	Frameshift	Hom	No	c.144delC	p.Leu49TrpfsTer23	0.00008749	Sperm donation
	Pt55	Teratozoopermia (MMAF)	*CFAP43* (NM_025145.5)	Splice site	Hom	NP	c.3541-2A > C	p.?	Not listed	ICSI
	Pt65	AT	*DNAH1* (NM_015512.4)	Stop-gain Frameshift Missense	Comp. Het	No	c.9610C > T c.6131del c.9777T > G	p.Arg3204 * p.Phe2044Serfs *13 p.Ser3259Arg	Not listed Not listed 0.000008037	ICSI
	Pt77	SPD (OAT)	*GALNTL5* (NM_145292.3)	Frameshift	Het	NP	c.153dup	p.Val52Serfs*23	Not listed	*
F	Pt2	POI	*HFM1* (NM_001017975.4)	Stop-gain	Hom	Yes	c.1905T > A	p.Tyr635*	Not listed	Oocyte Donation **
	Pt38	OOMD	*TUBB8* (NM_177987.2)	Missense	Hom	Yes	c.922G > A	p.Gly308Ser	Not listed	Oocyte donation
	Pt71	OOMD	*PATL2* (NM_001145112.1)	Stop-gain	Hom	Yes	c.478C > T	p.Arg160*	0.00003245	Oocyte donation

M: male, F: female, SPD: sperm production defect, MMAF: multiple morphological abnormalities of the sperm flagella, AT: astheno-teratozoospermia, OAT: oligo-astheno-teratozoospermia, POI: premature ovarian insufficiency, OOMD: oocyte maturation defect, Het: heterozygous, Hom: homozygous, Comp. Het: compound heterozygous, NP: not provided, ART option: options for assisted reproductive techniques, ICSI: intra-cytoplasmic sperm injection, *: further investigation needed, **: if ovarian reserve too low.

**Table 5:** Available gene panel publications in the infertility field.

Infertility Type	#Genes	#Cases (Male/ Female- Phenotype)	HTS Quality			Filtered-Out Frequency	Diagnostic Yield	References
			Mean Coverage	Depth of Coverage	% Targetted Bases			
Syndromic/ non-syndromic	284	48 idiopathic POI females	145X	10X	99.38%	> 0.1%	2%	[6]
Male infertility (genes based on mouse model)	175	33 idiopathic NOA	300X	NP	NP	> 5%	6.3%	[7]
Syndromic/ non-syndromic	75	17 female, 6 male with different infertility phenotype	180	20X	98%	> 5%	8.7%	[8]
Syndromic/ non-syndromic	9	241 idiopathic male infertility cases	351X	10X	93.5%	> 1%	0.4%	[9]
Syndromic/ non-syndromic	15	25 idiopathic male infertility with SPD	ND	ND	ND	ND	12%	[10]
Syndromic/ non-syndromic	110	22 male infertility cases	286–539X *	10X *	91.3–98% *	ND	25%	[11]
Non-syndromic	51	15 female, 79 male with different infertility phenotype	457X	30X	99.8%	>1%	8.5%	Present study

HTS: high-throughput sequencing. POI: primary ovarian insufficiency, NOA: non-obstructive azoospermia. Mean coverage: average number of reads that align to known reference bases. Depth of coverage: the number of unique reads that include a given nucleotide in the reconstructed sequence, also known as on-target read depth. % targeted bases: percentage of target bases that are successfully sequenced with a given depth of coverage. SPD: sperm production failure, oligozoospermia and non-obstructive azoospermia. NP: not provided. (*): quality data is given only for 5 positive cases.#: number.

## Data Availability

Data generated in this study are included in the following article and corresponding Supporting Information. The raw sequencing data generated in the course of this study are not publicly available due to the protocol and the corresponding consents used that did not include such information. All variants have been submitted to ClinVar (SCV001478453, SCV001478457, SCV001478463, SCV001478464, SCV001478466, SCV001478469, SCV001478470, SCV001478471) (https://www.ncbi.nlm.nih.gov/clinvar).

## Supplementary Materials

The following are available online at https://www.mdpi.com/2073-4425/12/3/410/s1, Figure S1: Visualization of HTS results for control samples C1 and C2 by the Integrative Genomics Viewer (IGV), Figure S2: HTS data visualized with the IGV for control sample C3 showing heterozygous *DPY19L2* gene deletion with point mutation in exon 8, Figure S3: Data visualization of HTS for control sample C4, Figure S4: A whole *DPY19L2* deletion in control sample C5. Table S1: Primers used for amplification of ten candidate variants selected in 8 genes after panel analysis, Table S2: Sequencing quality for each gene from the infertility panel, Table S3: Sequencing quality and bioinformatics analysis information of the 94 patients, Table S4: Previously identified mutations described in the literature for the 8 genes that we determined as mutated in this study, with corresponding zygosity and relevant references.
