# Supplementary material for: Evaluation of a Custom Design Gene Panel as a Diagnostic Tool for Human Non-Syndromic Infertility
Source: Genes (Basel). 2021 Mar 12;12(3):410. doi: 10.3390/genes12030410 (PMC7999157; doi:10.3390/genes12030410)
Supplement: Supplementary file 1 [file genes-12-00410-s001.pdf]

SUPPLEMENTARY MATERIALS

Figure S1: Visualization of HTS results for control samples C1 and C2 by The Integrative Genomics Viewer (IGV) (A) Sample C1 showing a no-stop mutation in *MAGEB4* (B) Sample C2 showing a stop-gain mutation in *TEX15*

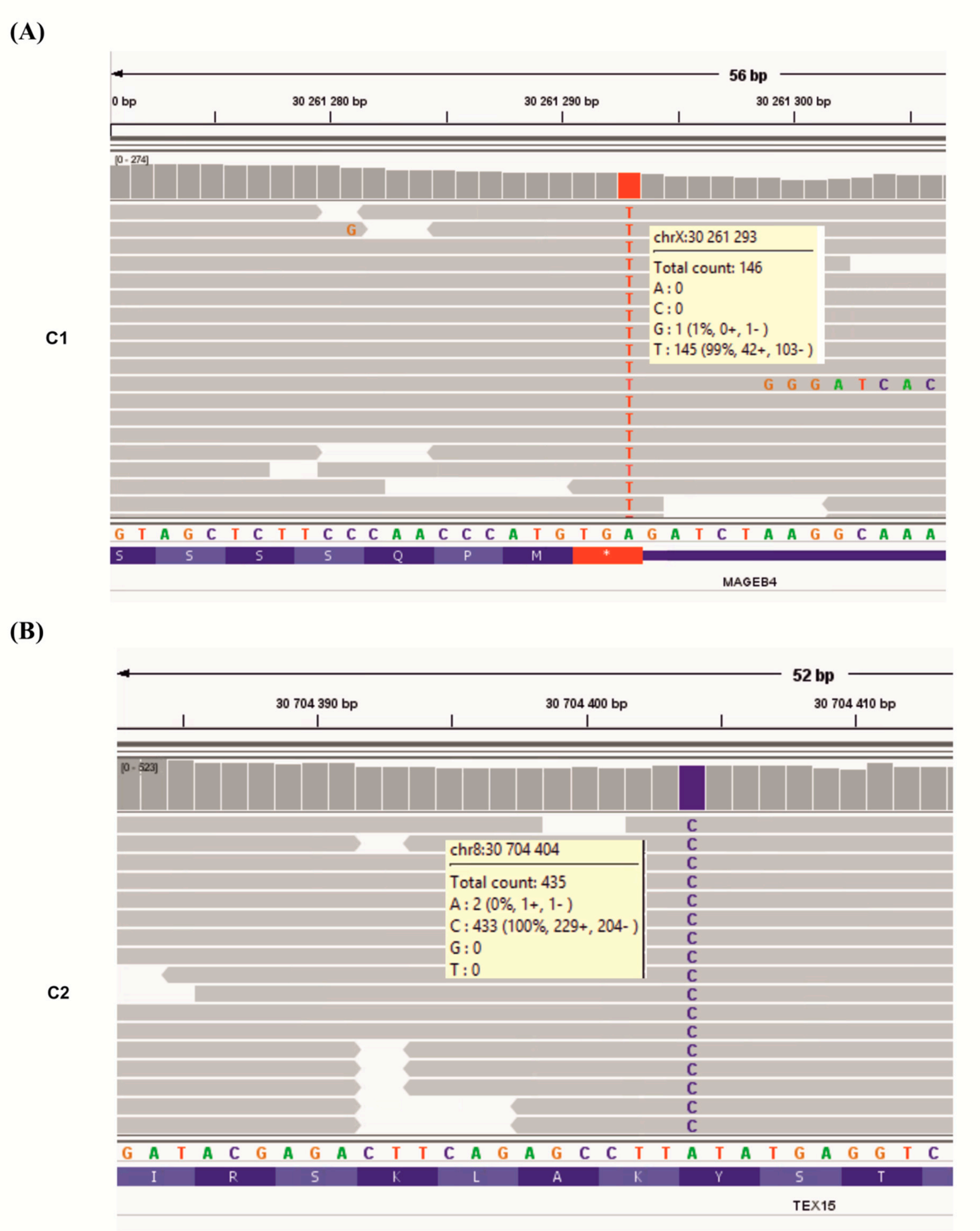

Figure S2: HTS data visualized with the IGV for control sample C3 showing heterozygous *DPY19L2* gene deletion with point mutation in exon 8 (A) *DPY19L2* gene sequencing data around exon 8 for sample C3 and a non-deleted sample for a control. There are 3149 reads in the non-deleted sample for the entire DPY19L2 gene in comparison to 1768 reads in sample C3, which is approximately half of the non-deleted samples indicating heterozygous *DPY19L2* gene deletion. Schematic representation of the mapped sequencing reads for the point mutation in exon 8 is shown in the enlarged area. (B) CANOES report which shows deletion in DPY19L2 for sample C3.

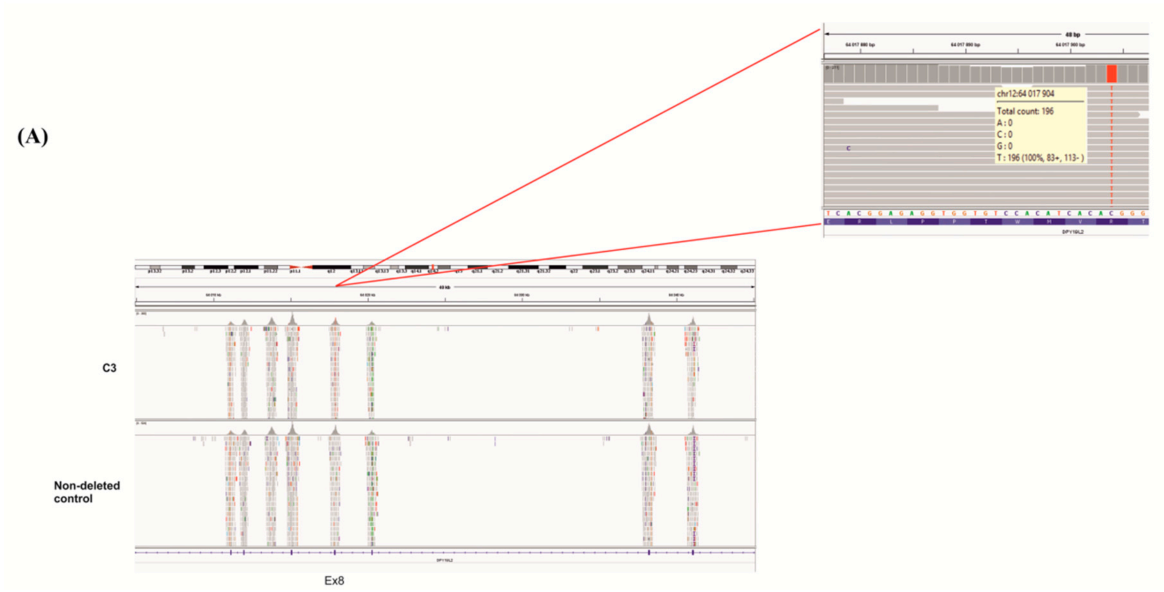

(B)

#Sex for sample C3 is: SEX_M									
#CNV chrom	CNV start	CNV end	CNV type	Sample ID	Gene name	NM	CDS length	tx lenth	location
12	63954266	64062198	DEL	C3	DPY19L2	NM_173812	2299	107933	exon1-exon22

Figure S3: Data visualization of HTS for control sample C4 (A) IGV plot showing the deletion of exon 5 and exon 6 of *DPY19L2* for sample C4, in comparison with non-deleted sample. (B) CANOES report also shows deletion of exon 5 and exon 6 in *DPY19L2* .

(A)

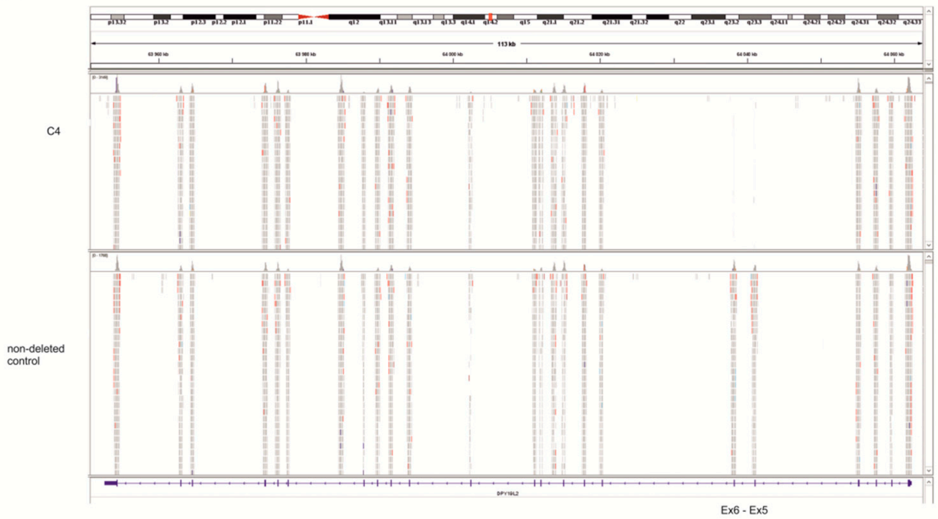

(B)

#Sex for sample C4 is: SEX_M									
#CNV chrom	CNV start	CNV end	CNV type	Sample ID	Gene name	NM	CDS length	tx lenth	location
12	64038157	64041170	DEL	C4	DPY19L2	NM_173812	217	3014	intron4-intron6

Figure S4: A whole *DPY19L2* deletion in control sample C5 (A) visualization of sequencing data of *DPY19L2* gene for sample C5 in comparison with non-deleted sample (B) CANOES report of C5 showing a whole *DPY19L2* gene deletion

(A)

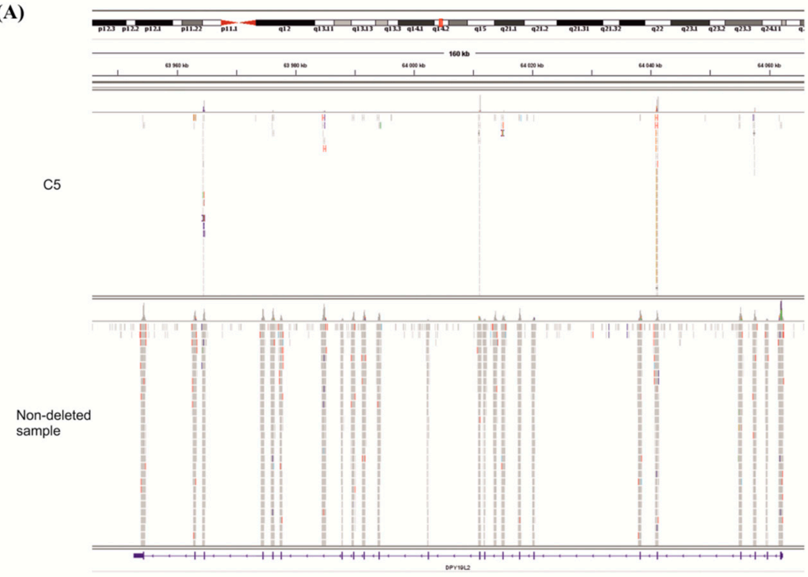

(B)

#Sex for sample C5 is: SEX_M									
#CNV chrom	CNV start	CNV end	CNV type	Sample ID	Gene name	NM	CDS length	tx lenth	location
12	63954266	64062198	DEL	C5	DPY19L2	NM_173812	2299	107933	exon1-exon22

Table S1: Primers used for amplification of ten candidate variants selected in 8 genes after panel analysis. PCR reactions were carried out with Qiagen Multiplex PCR kit (QIAGEN GmbH, Hilden, Germany), 35 cycles and in total 25µl volume

Gene name	Exon	Forward primer	Reverse primer	Annealing Temperature (°C)	PCR size product (bp)
PATL2	6-7	tgtccatcagagctgacctg	tcccacctgaacagacatga	64	623
AURKC	3	gactttccctccgcctaccctac	gctgggctcagacgtcaaaga	60	301
CFAP43	28	caagtgggagcatctctggt	atgaggagggagggtattgg	65	489
HFM1	16	aaaatgttcctgaagcaacaaag	ttttctctgctatattacttttaagcc	58	327
GALNTL5	2	agggccatcaacgtgattgt	agctctctgaagcaaatatagcca	64	634
KLHL10	3	agcaattcaaaggcagatcg	ccggctttgcattacttttc	60	906
DNAH1	38	cccacttacaggatgtgcag	tggattcctgcagggagtag	64	394
	60	cacccgaagtgggattagaa	ctcctaccaccctcttgctg	64	427
	62	aggccaacaaatggatcaag	tactgtggcctgagcatcac	64	361
TUBB8	4	gtgtgacgcttggctctttc	ttaaaacgcagcaggagatg	64	1264

Table S2: Sequencing quality for each gene from the Infertility Panel. Our diagnostic criteria require a sequencing depth of over 30 reads per base pair in at least 95% of all analyzed regions. 100% of coding exons were accurately sequenced in 40 genes; between 95 and 99.9% of coding exons were accurately sequenced in 11 genes

Gene name	RefSeq	Chromosome	#Coding Exon	%Coverage >30X
AKAP4	NM_003886.2	X	6	100
AURKC	NM_001015878.1	19	7	100
BMP15	NM_005448.2	X	2	100
BRDT	NM_001242806.2	1	18	100
CATSPER1	NM_053054.3	11	12	100
CCDC39	NM_181426.1	3	20	97.79
CFAP43	NM_025145.6	10	38	100
CFAP44	NM_001164496.1	3	34	97.73
DAX1(NROB1)	NM_000475.4	X	2	100
DNAH1	NM_015512.4	3	77	100
DNAH6	NM_001370.1	2	76	100
DPY19L2	NM_173812.4	12	22	100
FIGLA	NM_001004311.3	2	5	100
FMR1	NM_002024.5	X	17	100
FSHR	NM_000145.3	2	10	100
GALNTL5	NM_145292.3	7	8	100
GDF9	NM_005260.5	5	2	100
HFM1	NM_001017975.4	1	38	100
HIWI (PIWIL1)	NM_004764.4	12	20	100
HSF2	NM_004506.3	6	13	99.66
KLHL10	NM_152467.4	17	5	100
MAGEB4	NM_002367.3	X	1	100
MCM8	NM_001281521.1	20	18	99.13
MCM9	NM_017696.2	6	12	97.44
MEIOB	NM_001163560.2	16	13	95.57
MSH4	NM_002440.3	1	20	100
MTUS1	NM_001001924.2	8	14	95.59
NANOS1	NM_199461.3	10	1	100
NANOS3	NM_001098622.2	19	2	100
NOBOX	NM_001080413.3	7	10	100
NPAS2	NM_002518.3	2	20	100
NR5A1	NM_004959.4	9	6	100
PATL2	NM_001145112.1	15	15	100
PGRMC1	NM_006667.4	X	3	100
PICK1	NM_001039583.1	22	12	100
PLCZ1	NM_033123.3	12	14	100
SEPT12	NM_144605.4	16	9	100
SLC26A8	NM_001193476.1	6	19	100
SOHLH1	NM_001012415.2	9	7	97.92
SPAG17	NM_206996.3	1	48	100
SPATA16	NM_031955.5	3	10	100
SPINK2	NM_001271722.1	4	2	100
STAG3	NM_001282717.1	7	33	100
SYCE1	*NM_001143764.1*	10	13	100
TAF4B	NM_001293725.1	18	15	95.24
TEX11	NM_001003811.1	X	29	96.05
TEX14	NM_001201457.1	17	32	100
TEX15	NM_031271.3	8	4	100
TUBB8	NM_177987.2	10	4	100
Wt1	NM_024426.3	11	10	99.52
ZMYND15	NM_001267822.1	17	13	100

Table S3 : Sequencing quality and bioinformatics analysis information of the 94 patients. F :Female, M :Male, POI :Primary ovarian insufficiency, SPD:Sperm production defect, OOMD:oocyte maturation defect, OA: Oligoasthenozoospermia, AT:Asthenozoospermia, OT:Oligoteratozoospermia, OAT:Oligoasthenoteratozoospermia, CNV: copy number variations, DUP:Duplication, DEL: Deletions, Hom:homozygous, Het:heterozygous

Code	Sex	Clinical indication	Coverage (X)	30x(%)	Total #variants	# Filtered variations	# Variations related to phenotype	Mutation	Zygosity	Coding Effect	Gene name
**Pt1**	F	POI	652.41	99.9085	281	18	0	0			
**Pt2**	F	POI	585.05	99.9363	274	26	2	1	Hom	stop-gain	*HFM1*
**Pt3**	F	POI	652.45	99.9455	248	18	3	0			
**Pt4**	M	SPD	447.55	99.9663	330	20	5	0			
**Pt5**	M	SPD	652.1	99.993	293	20	7	0			
**Pt6**	M	SPD	493.2	99.9813	308	17	5	0			
**Pt7**	M	SPD	410.68	99.916	268	19	5	0			
**Pt8**	M	SPD	711.68	99.9101	314	25	9	0			
**Pt9**	M	SPD	695.67	99.9781	276	15	4	0			
**Pt10**	M	SPD	798.11	99.9791	300	24	5	0			
**Pt11**	M	SPD	685.53	99.9631	285	26	8	0			
**Pt12**	M	SPD	563.27	99.9524	315	22	10	1	Het	missense	*KLHL10*
**Pt13**	M	SPD	643.01	99.9936	227	20	3	0			
**Pt14**	M	SPD	623.99	99.9663	249	18	6	0			
**Pt15**	M	SPD	756.82	99.993	275	20	6	0			
**Pt16**	M	SPD	379.8	99.9048	297	16	5	0			
**Pt17**	M	SPD	633.17	99.992	303	21	6	0			
**Pt18**	M	SPD	772.57	99.9294	295	15	4	0			
**Pt19**	M	SPD	804.98	99.992	334	27	8	0			
**Pt20**	M	SPD	868.19	99.9807	320	25	6	0			
**Pt21**	M	SPD	905.33	99.9845	276	14	3	0			
**Pt22**	M	SPD	861.61	99.9631	314	23	7	0			

**Pt23**	M	SPD	689.16	99.9133	287	20	8	0			
**Pt24**	M	SPD	922.67	99.993	241	13	3	0			
**Pt25**	M	SPD	781.31	99.9914	282	20	5	0			
**Pt26**	M	SPD	852.86	99.9936	291	25	7	0			
**Pt27**	M	SPD	683.43	99.9679	259	24	7	0			
**Pt28**	M	SPD	782.95	100	283	30	8	0			
**Pt29**	M	SPD	799.33	99.9759	306	31	4	0			
**Pt30**	M	SPD	650.32	99.8936	297	26	9	0			
**Pt31**	F	POI	369.24	99.2966	290	13	1	0			
**Pt32**	F	POI	361.34	99.403	310	17	2	0			
**Pt33**	F	POI	283.58	99.7127	365	40	3	0			
**Pt34**	F	POI	264.95	99.6742	333	29	7	0			
**Pt35**	F	POI	308.89	99.8952	269	19	5	0			
**Pt36**	F	POI	393.42	99.9406	298	21	5	0			
**Pt37**	F	OOMD	280.39	99.5475	318	20	0	0			
**Pt38**	F	OOMD	258.23	99.2335	266	19	1	1	Hom	missense	*TUBB8*
**Pt39**	F	OOMD	346.39	99.9626	332	19	3	0			
**Pt40**	M	Teratozoospermia	305.08	99.6528	304	26	8	0			
**Pt41**	M	Teratozoospermia	402.61	99.9497	272	27	10	1	Hom	frameshift	*AURKC*
**Pt42**	M	SPD	264.27	99.7165	311	26	15	0			
**Pt43**	M	SPD	218.54	99.4506	272	22	9	0			
**Pt44**	M	SPD	252.03	99.6058	280	19	6	0			
**Pt45**	M	SPD	208.21	99.7577	319	15	9	0			
**Pt46**	M	SPD	295.59	99.877	272	26	12	0			
**Pt47**	M	SPD	294.12	99.8219	325	28	6	0			
**Pt48**	M	SPD	329.02	99.587	332	23	10	0			
**Pt49**	M	SPD	262.25	99.6058	270	20	8	0			

**Pt50**	M	SPD	323.52	99.8379	302	30	14	0			
**Pt51**	M	SPD	237.59	99.7058	329	30	12	0			
**Pt52**	M	Asthenozoospermia	183.91	98.6755	307	23	2	0			
**Pt53**	M	SPD	286.69	99.8235	329	40	18	0			
**Pt54**	M	SPD	324.74	99.8155	269	31	6	0			
**Pt55**	M	Teratozoospermia	300.88	99.8481	298	27	10	1	Hom	splice-site	*CFAP43*
**Pt56**	M	AT	323.38	99.8245	297	23	13	0			
**Pt57**	M	Teratozoospermia	350.41	99.8171	257	13	5	0			
**Pt58**	M	Teratozoospermia	358.86	99.8978	306	25	5	0			
**Pt59**	M	AT	420	99.9497	310	21	8	0			
**Pt60**	M	OAT	317.91	99.9315	312	20	14	0			
**Pt61**	F	POI	419.89	99.9187	287	16	1	0			
**Pt62**	M	SPD	324.78	99.4121	284	17	6	0			
**Pt63**	M	SPD	416.1	99.8064	255	12	7	0			
**Pt64**	M	SPD	275.2	99.6475	269	21	10	0			
**Pt65**	M	AT	378.91	99.9636	283	21	8	3	Het	stop-gain	*DNAH1*
									Het	frameshift	
									Het	missense	
**Pt66**	M	OAT	337.08	99.9321	286	28	26	0			
**Pt67**	F	POI	402.92	99.9733	269	18	3	0			
**Pt68**	M	Teratozoospermia	424.96	99.9797	289	24	7	0			
**Pt69**	M	OT	448.57	99.7368	277	29	22	0			
**Pt70**	M	SPD	439.7	99.985	264	20	11	0			
**Pt71**	F	OOMD	388.86	99.7641	310	18	3	1	Hom	stop-gain	*PATL2*
**Pt72**	M	OAT	330.95	99.7919	244	12	6	0			
**Pt73**	M	Teratozoospermia	379.2	99.9872	250	26	4	0			
**Pt74**	M	OAT	337.81	99.8278	279	19	14	0			

**Pt75**	M	OAT	341.86	99.839	346	27	14	0			
**Pt76**	M	OAT	301.18	99.7801	281	16	13	0			
**Pt77**	M	OAT	369.18	99.9716	267	26	19	1	Het	frameshift	*GALNTL5*
**Pt78**	M	OAT	473.53	99.9711	297	33	21	0			
**Pt79**	M	SPD	403.85	99.9449	292	14	8	0			
**Pt80**	M	SPD	531.58	99.9412	254	23	10	0			
**Pt81**	M	SPD	425.04	99.9438	291	28	15	0			
**Pt82**	M	SPD	325.35	99.8871	263	20	7	0			
**Pt83**	M	SPD	383.32	99.9551	276	22	12	0			
**Pt84**	M	OA	444.95	99.908	304	16	11	0			
**Pt85**	M	SPD	541.4	99.9106	320	31	11	0			
**Pt86**	M	SPD	451.98	99.8026	269	12	7	0			
**Pt87**	M	OAT	443.05	99.8754	268	25	18	0			
**Pt88**	M	OAT	420.11	99.9128	355	34	28	0			
**Pt89**	M	OAT	365.09	99.7892	275	19	15	0			
**Pt90**	M	OAT	502.81	99.9337	309	26	19	0			
**Pt91**	M	OAT	468.43	99.9604	299	18	12	0			
**Pt92**	M	OAT	487.56	99.9139	374	30	25	0			
**Pt93**	M	OAT	528.62	99.8973	361	40	28	0			
**Pt94**	M	OAT	455.93	99.9005	272	15	10	0			

Table S4: Previously identified mutations described in the literature for the 8 genes that we determined as mutated in this study, with corresponding zygosity and relevant references. Heterozygous mutations that exists with another heterozygous mutation in patients indicated by using asterisk(*)

AURKC : NM_001015878.1

	c.DNA change	Protein change	Zygosity	References
**1**	c.144delC	p.Leu49Trpfs*23	Homozygous<br>Heterozygous*	Dieterich et al 2007, Harbuz et al 2009<br>Dieterich et al 2009, Ben Khelifa et al 2011
**2**	c.269G>A	p.Arg90Gln	Homozygous	Hua and Wan 2018
**3**	c.382C>T	p.Leu128Phe	Heterozygous*	Bai et al 2020
**4**	c.436-2A>G	p. ?	Heterozygous*	Ben Khelifa et al 2011
**5**	c.572C>T	p. Thr191Ile	Heterozygous*	Bai et al 2020
**6**	c.686G>A	p.Cys229Tyr	Heterozygous*	Harbuz et al 2009, Dieterich et al 2009
**7**	c.744C>G	p.Tyr248*	Homozygous	Ben Khelifa et al 2012, Ortega et al 2019

CFAP43 : NM_025145.6

	c.DNA change	Protein change	Zygosity	References
**1**	c.114_117delCACC	p.Asn38Lysfs*10	Heterozygous*	Coutton et al 2019
**2**	c.120_121delTT	p.Ile40Metfs*12	Heterozygous*	Coutton et al 2019
**3**	c.253C>T	p.Arg85Trp	Heterozygous*	Tang et al 2017
**4**	c.386 C>A	p.Ser129Tyr	Heterozygous*	Tang et al 2017
**5**	c.739A>T	p.Lys247*	Homozygous	Wu et al 2019
**6**	c.1040T>C	p.Val347Ala	Heterozygous*	Coutton et al 2019
**7**	c.1140_1143delTAAA	p.Asn380Lysfs*3	Homozygous	Wu et al 2019
**8**	c.1240_1241delGT	p.Val414Leufs*46	Homozygous	Coutton et al 2019
**9**	c.1302dupT	p.Leu435Serfs*26	Heterozygous*	Coutton et al 2019
**10**	c.1474G>C	p.Gln492Arg	Homozygous	Wu et al 2019
**11**	c.2141+5G>A	p. ?	Homozygous	Coutton et al 2019
**12**	c.2658G>A	p.Trp886*	Homozygous	Coutton et al 2019
**13**	c.2680C>T	p.Arg894*	Homozygous	Coutton et al 2019
**14**	c.2802T>A	p.Cys934*	Heterozygous**	Tang et al 2017
**15**	c.3352C>T	p.Arg1118*	Homozygous	Coutton et al 2019
**16**	c.3374delG	p.Gly1125Glufs*12	Homozygous	Coutton et al 2019
**17**	c.3541-2A>C	p.Ser1181Lysfs*4	Homozygous	Coutton et al 2019
**18**	c.3661-2delA	p.Glu1221_Lys1256del	Homozygous<br>Homozygous	Li et al 2020<br>Sha et al 2019
**19**	c.3882delA	p.Glu1294Aspfs*47	Homozygous	Coutton et al 2019
**20**	c.3945_4431del	DEL ex32-34<br>(p.Ile1316Leufs*10)	Heterozygous*	Tang et al 2017
**21**	c.4132C>T	p.Arg1378*	Heterozygous*	Tang et al 2017
**22**	c.4600C>G	p.Leu1534Val	Homozygous	Wu et al 2019

GALNTL5 : NM_145292.3

	c.DNA change	Protein change	Zygosity	References
**1**	c.369-2A>G	p. ?	Heterozygous	Hagiuda et al 2019*
**2**	c.764delT	p.Leu255Argfs*2	Heterozygous	Takasaki et al 2014

*Identified in sperm

KLHL10 : NM_152467.4

	c.DNA change	Protein change	Zygosity	References
1	c.242A>T	p.Asn81Ile	Heterozygous<br>Heterozygous	Araujo et al 2019<br>Rocco et al 2020
2	c.647A>C	p.Gln216Pro	Heterozygous	Yatsenko et al 2006
3	c.887T>C	p.Ile296Thr	Heterozygous<br>Heterozygous	Araujo et al 2019<br>Rocca et al 2019
4	c.937G>A	p.Ala313Thr	Heterozygous	Yatsenko et al 2006
5	c.1302+123_1302+126delTCTT	p. ?	Heterozygous	Yatsenko et al 2006

DNAH1 : NM_015512.4

	c.DNA change	Protein change	Zygosity	References
1	c.2127dupC	p.Ile710Hisfs*4	Homozygous	Coutton et al 2018
2	c.2610G>A	p.Trp870*	Heterozygous *	Tang et al 2017, Sha et al 2017, Liu et al 2019
3	c.3108G>A	p.Trp1036*	Heterozygous*	Tang et al 2017, Sha et al 2017, Liu et al 2019
4	c.3593_3593delA	p.Asp1198Alafs*6	Heterozygous*	Tang et al 2017
5	c.3836A>G	p.Lys1279Arg	Heterozygous*	Sha et al 2017, Liu et al 2019
6	c.3846C>G	p.Tyr1282*	Homozygous	Liu et al 2019
7	c.3860T>G	p.Val1287Gly	Homozygous	Amiri-Yekta et al 2016
8	c.3877G>A	p.Asp1293Asn	Homozygous	Ben Khalifa et al 2014
9	c.3931C>T	p.Gln1311*	Homozygous	Coutton et al 2019
10	c.4115C>T	p.Thr1372Met	Heterozygous*	Tang et al 2017, Sha et al 2017, Liu et al 2019
11	c.4531G>A	p.Val1511Met	Heterozygous	Coutton et al 2018
12	c.4642C>G	p.Leu1548Val	Heterozygous	Coutton et al 2018
13	c.4744_4752delCCAGCTGG C	p.Pro1582_Gly1584del	Homozygous	Coutton et al 2018
14	c.4983G>A	p.Val1661=	Homozygous	Coutton et al 2019
15	c.5094+1G>A	NA	Homozygous	Ben Khalifa et al 2014
16	c.5104A>T	p.Arg1702*	Heterozygous*	Tang et al 2017
17	c.5105G>C	p.Arg1702Pro	Heterozygous *	Tu et al 2019
18	c.5094+1G>A	NA	Homozygous	Ben Khalifa et al 2014
19	c.5584G>A	p.Gly1862Arg	Heterozygous*	Tang et al 2017
20	c.5626G>C	p.Ala1876Pro	Heterozygous *	Yang et al 2018, Liu et al 2019
21	c.5766-2A>G	NA	Heterozygous*	Sha et al 2017, Liu et al 2019
22	c.5864G>A	p.Trp1955*	Heterozygous*	Tang et al 2017, Sha et al 2017, Liu et al 2019
23	c.6212T>G	p.Leu2071Arg	Heterozygous*	Tang et al 2017, Sha et al 2017, Liu et al 2019
24	c.6253_6254del	p.Arg2085fs	Heterozygous*	Sha et al 2017, Liu et al 2019
25	c.6257_6258delAG	p.Glu2086Glyfs*8	Heterozygous*	Tang et al 2017
26	c.6448G>A	p.Val2150Met	Heterozygous*	Tang et al 2017
27	c.6822C>G	p.Asp2274Glu	Heterozygous*	Sha et al 2017, Liu et al 2019
28	c.7066C>T	p.Arg2356Trp	Heterozygous	Sha et al 2017, Yang et al 2018
29	c.7153T>A	p.Trp2385Arg	Heterozygous *	Coutton et al 2018
30	c.7377+1G>C	NA	Homozygous	Sha et al 2017, Liu et al 2019
31	c.7378+1G>C	NA	Homozygous	Tang et al 2017
32	c.7396C>T	p.Arg2466*	Heterozygous*	Tang et al 2017
33	c.7471C>A	p.Gln2491Lys	Heterozygous *	Li et al 2019
34	c.7531delC	p.Gln2511Serfs*27	Homozygous	Coutton et al 2018
35	c.7864C>T	p.Arg2622*	Heterozygous*	Tang et al 2017
36	c.8151-1G>C	NA	Homozygous<br>Heterozygous*	Liu et al 2019<br>Tu et al 2019
37	c.8322+3del	NA	Heterozygous*	Yang et al 2018
38	c.8626-1G>A	NA	Homozygous	Amiri-Yekta et al 2016, Coutton et al 2019

**39**	c.8811+1G>T	NA	Heterozygous*	Tang et al 2017
**40**	c.9505C>G	p.Arg3169Gly	Heterozygous*	Coutton et al 2018, Liu et al 2019
**41**	c.9567delC	p.Glu3190Serfs*4	Homozygous	Coutton et al 2019
**42**	c.9621+1G>C	NA	Homozygous	Coutton et al 2019
**43**	c.9661G>A	p.Gly3221Arg	Heterozygous*	Tang et al 2017
**44**	c.9685C>T	p.Arg3229Cys	Heterozygous*	Tang et al 2017
**45**	c.9850G>A	p.Glu3284Lys	Heterozygous*	Sha et al 2017, Liu et al 2019
**46**	c.10464_10467delAGAG	p.Arg3490Glnfs*4	Heterozygous*	Tang et al 2017
**47**	c.10630G>T	p.Glu3544*	Heterozygous*	Sha et al 2017, Liu et al 2019
**48**	c.11213G>A	p.Trp3738*	Heterozygous*	Tang et al 2017
**49**	c.11726_11727delCT	p.Pro3909Argfs*33	Homozygous<br>Heterozygous *	Wang et al 2017<br>Yang et al 2018, Tang et al 2017, Sha et al 2017, Liu et al 2019, Tu et al 2019
**50**	c.11779G>A	p.Asp3927Asn	Heterozygous *	Liu et al 2019
**51**	c.11787_11787+1insT	NA	Heterozygous*	Tang et al 2017
**52**	c.11788-1G>A	NA	Homozygous	Ben Khalifa et al 2014
**53**	c.12200_12202del	p.4067_4068del	Heterozygous *	Sha et al 2017, Liu et al 2019
**54**	c.12200_12202delACA	p.Asn4068del	Heterozygous*	Tang et al 2017
**55**	c.12286C>T	p.Arg4096Cys	Heterozygous *	Tu et al 2019
**56**	c.12287G>T	p.Arg4096Leu	Heterozygous*	Tang et al 2017, Sha et al 2017, Liu et al 2019
**57**	c.12397C>T	p.Arg4133Cys	Heterozygous*	Tang et al 2017, Sha et al 2017, Liu et al 2019
**58**	c.12520G>A	p.Ala4174Thr	Homozygous	Tang et al 2017
**59**	c.12796T>C	p.Ter4266Glnext*21	Homozygous	Ben Khalifa et al 2014

PATL2 : NM_001145112.1

	**c.DNA change**	**Protein change**	**Zygosity**	**References**
**1**	c.223-14_223-2del13	p.Arg75Valfs*21	Heterozygous *	Chen et al 2017a, Huang 2018, Wu 2019
**2**	c.478C>T	p.Arg160*	Homozygous	Maddirevula et al 2017, Christou-Kent et al 2018
**3**	c.558T>A	p.Tyr186*	Heterozygous*	Chen et al 2017a
**4**	c.566T>G	p.Leu189Arg	Heterozygous*	Chen et al 2017a
**5**	c.649T>A	p.Tyr217Asn	Heterozygous*	Chen et al 2017a
**6**	c.716delA	p.Asn239Thrfs*9	Heterozygous*	Wu et al 2019
**7**	c.778G>A	p.Val260Met	Heterozygous*	Wu et al 2019
**8**	c.784C>T	p.Arg262*	Homozygous	Chen et al 2017a
**9**	c.839G>A	p.Arg280Gln	Heterozygous*	Chen et al 2017a
**10**	c.865delA	p.Thr289Leufs*6	Heterozygous*	Huang et al 2018
**11**	c.877G>T	p.Asp293Tyr	Heterozygous*	Wu et al 2019
**12**	c.898C>T	p.Gln300*	Heterozygous*	Wu et al 2019
**13**	c.953T>C	p.Ile318Thr	Heterozygous*	Chen et al 2017a
**14**	c.1108G>A	p.Gly370Arg	Homozygous	Maddirevula et al 2017
**15**	c.1127G>A	p.Arg376Gln	Homozygous	Huang et al 2018
**16**	c.1225-2A>G	p. ?	Heterozygous*	Huang et al 2018
**17**	c.1225+2T>C	p. ?	Heterozygous*	Chen et al 2017a
**18**	c.1273A>C	p.Thr425Pro	Heterozygous*	Wu et al 2019
**19**	c.1282G>T	p.Glu428*	Heterozygous*	Huang et al 2018
**20**	c.1300C>T	p.Gln434*	Heterozygous*	Huang et al 2018
**21**	c.1376C>A	p.Ser459Tyr	Homozygous	Liu et al 2020
**22**	c.1528C>A	p.Pro510Thr	Homozygous	Liu et al 2020

HFM1 :NM_001017975.4

	c.DNA change	Protein change	Zygosity	References
1	c.1006 + 1G > T	p. ?	Heterozygous*	Liu et al 2020
2	c.1241A>C	p.His414Pro	Heterozygous	Pu et al 2016
3	c.1686-1G>C	p. ?	Heterozygous*<br>Heterozygous	Wang et al 2014<br>Pu et al 2016
4	c.2206G>A	p.Gly736Ser	Heterozygous*	Wang et al 2014
5	c.2325C>A	p.Phe775Leu	Heterozygous	Pu et al 2016
6	c.2651T>G	p.Ile884Ser	Heterozygous*	Wang et al 2014
7	c.3100G > A	p.Gly1034Ser	Heterozygous*	Liu et al 2020
8	c.3470G>A	p.Cys1157Tyr	Heterozygous	Zhe et al 2019
9	c.3580C>T	p.Arg1194Cys	Heterozygous	Pu et al 2016
10	c.3929_3930delinsG	p.Pro1310Argfs*41	Heterozygous*	Wang et al 2014
11	c.4061delC	p.Pro1354Leufs*71	No info	Yanus et al 2019

TUBB8 : NM_177987.2

	c.DNA change	Protein change	Zygosity	References
1	c.5G>A	p.Arg2Lys	Heterozygous	Feng et al 2016a
2	c.5G>T	p.Arg2Met	Heterozygous	Huang et al 2017,Chen et al 2017b
3	c.10A>C	p.Ile4Leu	Heterozygous	Chen et al 2017b, Chen et al 2019
4	c.12C>G	p.Ile4Met	Heterozygous	Zhao et al 2020
5	c.35G>A	P.Cys12Tyr	Homozygous	Chen et al 2017b
6	c.107G>A	p.Gly17Ser	Heterozygous	Zhao et al 2020
7	c.80_100del21	p.Glu27_Ala33del	Homozygous<br>Heterozygous*	Feng et al 2016b<br>Zhao et al 2020
8	c.140C>T	p.His28Tyr	Heterozygous*	Zhao et al 2020
9		p.Arg46Cys		Yang et al 2021
10	c.206T>C	p.Tyr50His	Heterozygous	Zhao et al 2020
11	c.148_154delins<br>CACCACCACGAGGCC<br>AGCGGTGCGACCCCC<br>GTCCTTCCCCCACCCA<br>ACGTGCACCACC	p.Tyr50_Asn52delins<br>HHHESGATPVLPPP<br>NVHHH	Heterozygous*	Zhao et al 2020
12	c.161C>T	p. A54V	Homozygous	Xing et al 2020
13	c.178G>C	p.Val60Leu	Heterozygous	Zhao et al 2020
14	c.181C>A	p.Pro61Thr	Heterozygous	Chen et al 2019
15	c.209C>T	p.Pro70Leu	Heterozygous	Chen et al 2017b
16	c.292G>A	p.Gly79Glu	Heterozygous	Zhao et al 2020
17	c.305C>T	p.Pro87Leu	Heterozygous	Zhao et al 2020
18	c.292G>A	p.Gly98Arg	Heterozygous	Wang et al 2018, Chen et al 2019
19	c.351G>A	p.Gly98Glu	Heterozygous	Zhao et al 2020
20	c.322G>A	p.Glu108Lys	Homozygous<br>Heterozygous *	Yuan et al 2018<br>Chen et al 2019
21	c.411A>G	p.Asp118Gly	Heterozygous*	Zhao et al 2020
22	c.363_366delAAAG	p.Lys122Argfs*13	Heterozygous	Christou-Kent et al 2018
23	c.367G>A	p.Glu123Lys	Heterozygous	Yang et al 2021
24	c.382dup	p.Asp128Glyfs*27	Heterozygous*	Zhao et al 2020
25	c.394G>T	p.Gly132Cys	Heterozygous	Yang et al 2021
26	c.456T>C	p.Phe133Ser	Heterozygous*	Zhao et al 2020
27	c.458C>T	p.Gln134*	Heterozygous*	Zhao et al 2020
28	c.422G>C	p.Gly141Ala	Heterozygous	Yang et al 2021
29	c.426dupG	p.Thr143Aspfs*12	Homozygous<br>Heterozygous *	Feng et al 2016b<br>Chen et al 2019

**30**	c.552C>T	p.Thr166Ile	Heterozygous	Zhao et al 2020
**31**	c.523G>A	p.Val175Met	Heterozygous	Chen et al 2019
**32**	c.527C>T	p.Ser176Leu	Heterozygous	Feng et al 2016a ; Feng et al 2016b, Chen et al 2019
**33**	c.535G>A	p.Val179Met	Heterozygous	Huang et al 2017
**34**	c.593G>T	p.Val179Leu	Heterozygous	Zhao et al 2020
**35**	c.539T>C	p.Val180Ala	Heterozygous	Yang et al 2021
**36**	c.544C>T	p.Pro182Ser	Heterozygous	Yang et al 2021
**37**	c.550A>G	p.Asn184Asp	Heterozygous	Yang et al 2021
**38**	c.580G>A	p.Glu194Lys	Heterozygous*	Chen et al 2017b
**39**	c.594G>C	p.Glu198Asp	Heterozygous	Yang et al 2021
**40**	c.600T>G	p.Phe200Leu	Heterozygous	Chen et al 2019
**41**	c.608A>G	p.Asp203Gly	Heterozygous	Zhao et al 2020
**42**	c.613G>A	p.Glu205Lys	Heterozygous	Chen et al 2017b
**43**	c.628A>G	p.Ile210Val	Heterozygous	Feng et al 2016b
**44**	c.629T>A	p.Ile210Lys	Heterozygous	Yang et al 2021
**45**	c.720C>T	p.Thr221Ile	Heterozygous	Zhao et al 2020
**46**	c.686T>C	p.Val229Ala	Heterozygous	Feng et al 2016a
**47**	c.713C>T	p.Thr238Met	Heterozygous	Feng et al 2016b
**48**	c.715T>A	p.Cys239Ser	Heterozygous	Zhao et al 2020
**49**	c.721C>T	p.Arg241Cys	Homozygous	Chen et al 2019
**50**	c.722G>A	p.Arg241His	Heterozygous	Chen et al 2019
**51**	c.728C>T	p.Pro243Leu	Heterozygous	Zhao et al 2020
**52**	c.728C>G	p.Pro243Arg	Heterozygous	Zhao et al 2020
**53**	c.735G>C	p.Gln245His	Heterozygous	Chen et al 2019
**54**	c.736C>G	p.Leu246Val	Heterozygous	Zhao et al 2020
**55**	c.743C>T	p.Ala248Val	Heterozygous	Zhao et al 2020
**56**	c.763G>A	p.Val255Met	Heterozygous	Feng et al 2016b, Chen et al 2019
**57**	c.784C>T	p.Arg262Trp	Heterozygous	Feng et al 2016b
**58**	c.785G>A	p.Arg262Gln	Heterozygous	Feng et al 2016a
**59**	c.845G>C	p.Arg282Pro	Heterozygous	Zhao et al 2020
**60**	c.853A>C	p.Thr285Pro	Heterozygous	Feng et al 2016b
**61**	c.944G>C	p.Asp296His	Heterozygous	Chen et al 2019
**62**	c.950A>G	p.Asn298Ser	Heterozygous	Zhao et al 2020
**63**	c.958G>A	p.Met300Ile	Heterozygous	Feng et al 2016a
**64**	c.925C>T	p.Arg309Cys	Heterozygous	Zhao et al 2020
**65**	c.940G>T	p.Ala314Ser	Heterozygous	Zhao et al 2020
**66**	c.990G>A	p.Met330Ile	Heterozygous	Chen et al 2017b
**67**	c.1000C>G	p.Gln334Glu	Heterozygous	Chen et al 2019
**68**	c.1043A>G	p.Asn348Ser	Heterozygous	Feng et al 2016b
**69**	c.1054G>T	p.Ala352Ser	Heterozygous	Xiang et al 2018
**70**	c.1057G>A	p.Val353Ile	Heterozygous	Chen et al 2017b ; Chen et al 2019
**71**	c.1061G>A	p.Cys354Tyr	Heterozygous	Chen et al 2019
**72**	c.1072C>G	p.Pro358Ala	Heterozygous	Chen et al 2019
**73**	c.1073C>T	p.Pro358Leu	Heterozygous	Chen et al 2019
**74**	c.1076G>A	p.Arg359Gln	Homozygous	Zhao et al 2020
**75**	c.1088T>C	p.Met363Thr	Heterozygous	Feng et al 2016a
**76**	c.1099T>C	p.Phe367Leu	Heterozygous	Chen et al 2019
**77**	c.1103T>C	p.Ile368Thr	Heterozygous*	Zhao et al 2020
**78**	c.1130T>C	p.Leu377Pro	Heterozygous	Zhao et al 2020
**79**	c.1139G>A	p.Arg380His	Heterozygous	Zhao et al 2020
**80**	c.1163T>C	p.Met388Thr	Heterozygous	Zhao et al 2020
**81**	c.1171C>T	p.Arg391Cys	Heterozygous	Chen et al 2019
**82**	c.1172G>A	p.Arg391His	Heterozygous	Zhao et al 2020
**83**	c.1178C>A	p.Ala393Asp	Heterozygous	Yang et al 2021

**84**	c.1187A>G	p.His396Arg	Heterozygous	Zhao et al 2020
**85**	c.1189T>G	p.Trp397Glu	Heterozygous	Yang et al 2021
**86**	c.1203_1204insCT	p.G402Lfs*15	Heterozygous	Yang et al 2021
**87**	c.1205dupG	p.Met403Hisfs*3	Homozygous	Chen et al 2019
**88**	c.1228G>A	p.Glu410Lys	Heterozygous	Chen et al 2019
**89**	c.1242C>G	p.Asn414Lys	Heterozygous	Yang et al 2021
**90**	c.1245G>A	p.Met415Ile	Heterozygous*	Chen et al 2017b
**91**	c.1249G>T	p.Asp417Tyr	Heterozygous	Chen et al 2019
**92**	c.1249G>A	p.Asp417Asn	Heterozygous	Feng et al 2016a
**93**	c.1270C>T	p.Gln424*	Homozygous	Chen et al 2019
**94**	c.1271A>G	p.Gln424Arg	Heterozygous	Yang et al 2021
**95**	c.736C>G	p.Leu426Val	Heterozygous	Zhao et al 2020
**96**	c.1286C>T	p.Thr429Met	Heterozygous*	Chen et al 2019
**97**	c.1301_1327del	p.E434_E442del	Heterozygous*	Chen et al 2019
**98**	ND	Exon1-4 del	Homozygous	Chen et al 2017b

SUPPLEMENTARY REFERENCES

Araujo TF, Friedrich C, Grangeiro CHP, Martelli LR, Grzesiuk JD, Emich J, Wyrwoll MJ, Kliesch S, Simões AL, Tüttelmann F. Sequence analysis of 37 candidate genes for male infertility: challenges in variant assessment and validating genes. *Andrology* 2020;**8**:434–441.

Amiri-Yekta A, Coutton C, Kherraf Z-E, Karaouzène T, Le Tanno P, Sanati MH, Sabbaghian M, Almadani N, Sadighi Gilani MA, Hosseini SH, *et al.* Whole-exome sequencing of familial cases of multiple morphological abnormalities of the sperm flagella (MMAF) reveals new DNAH1 mutations. *Hum Reprod* 2016;**31**:2872–2880.

Bai S, Hu X, Zhao Y, Li W, Wan Y, Jin R, Wang Y, Guo T, Tong X, Xu B. Compound heterozygosity for novel AURKC mutations in an infertile man with macrozoospermia. *Andrologia* 2020;e13663.

Ben Khelifa M, Zouari R, Harbuz R, Halouani L, Arnoult C, Lunardi J, Ray PF. A new AURKC mutation causing macrozoospermia: implications for human spermatogenesis and clinical diagnosis. *Mol Hum Reprod* 2011;**17**:762–768.

Ben Khelifa M, Coutton C, Blum MGB, Abada F, Harbuz R, Zouari R, Guichet A, May-Panloup P, Mitchell V, Rollet J, *et al.* Identification of a new recurrent aurora kinase C mutation in both European and African men with macrozoospermia. *Hum Reprod* 2012;**27**:3337–3346.

Ben Khelifa M, Coutton C, Zouari R, Karaouzène T, Rendu J, Bidart M, Yassine S, Pierre V, Delaroche J, Hennebicq S, *et al.* Mutations in DNAH1, which encodes an inner arm heavy chain dynein, lead to male infertility from multiple morphological abnormalities of the sperm flagella. *Am J Hum Genet* 2014;**94**:95–104.

Chen B, Zhang Z, Sun X, Kuang Y, Mao X, Wang X, Yan Z, Li B, Xu Y, Yu M, *et al.* Biallelic Mutations in PATL2 Cause Female Infertility Characterized by Oocyte Maturation Arrest. *Am J Hum Genet* 2017a;**101**:609–615.

Chen B, Li B, Li D, Yan Z, Mao X, Xu Y, Mu J, Li Q, Jin L, He L, *et al.* Novel mutations and structural deletions in TUBB8: expanding mutational and phenotypic spectrum of patients with arrest in oocyte maturation, fertilization or early embryonic development. *Hum Reprod* 2017b;**32**:457–464.

Chen B, Wang W, Peng X, Jiang H, Zhang S, Li D, Li B, Fu J, Kuang Y, Sun X, *et al.* The comprehensive mutational and phenotypic spectrum of TUBB8 in female infertility. *Eur J Hum Genet* 2019;**27**:300–307.

Christou-Kent M, Kherraf Z-E, Amiri-Yekta A, Le Blévec E, Karaouzène T, Conne B, Escoffier J, Assou S, Guttin A, Lambert E, *et al.* PATL2 is a key actor of oocyte maturation whose invalidation causes infertility in women and mice. *EMBO Mol Med* 2018;**10**:.

Coutton C, Vargas AS, Amiri-Yekta A, Kherraf Z-E, Ben Mustapha SF, Le Tanno P, Wambergue-Legrand C, Karaouzène T, Martinez G, Crouzy S, *et al.* Mutations in CFAP43 and CFAP44 cause male infertility and flagellum defects in Trypanosoma and human. *Nat Commun* [Internet] 2018;**9**:.

Coutton C, Martinez G, Kherraf Z-E, Amiri-Yekta A, Boguenet M, Saut A, He X, Zhang F, Cristou-Kent M, Escoffier J, *et al.* Bi-allelic Mutations in ARMC2 Lead to Severe Astheno-Teratozoospermia Due to Sperm Flagellum Malformations in Humans and Mice. *Am J Hum Genet* 2019;**104**:331–340.

Dieterich K, Soto Rifo R, Faure AK, Hennebicq S, Ben Amar B, Zahi M, Perrin J, Martinez D, Sèle B, Jouk P-S, *et al.* Homozygous mutation of AURKC yields large-headed polyploid spermatozoa and causes male infertility. *Nat Genet* 2007;**39**:661–665.

Dieterich K, Zouari R, Harbuz R, Vialard F, Martinez D, Bellayou H, Prisant N, Zoghmar A, Guichaoua MR, Koscinski I, *et al.* The Aurora Kinase C c.144delC mutation causes meiosis I arrest in men and is frequent in the North African population. *Hum Mol Genet* 2009;**18**:1301–1309.

Feng R, Sang Q, Kuang Y, Sun X, Yan Z, Zhang S, Shi J, Tian G, Luchniak A, Fukuda Y, *et al.* Mutations in TUBB8 and Human Oocyte Meiotic Arrest. *N Engl J Med* 2016a;**374**:223–232.

Feng R, Yan Z, Li B, Yu M, Sang Q, Tian G, Xu Y, Chen B, Qu R, Sun Z, *et al.* Mutations in TUBB8 cause a multiplicity of phenotypes in human oocytes and early embryos. *J Med Genet* 2016b;**53**:662–671.

Hagiuda J, Takasaki N, Oya M, Ishikawa H, Narimatsu H. Mutation of GALNTL5 gene identified in patients diagnosed with asthenozoospermia. *Hum Fertil (Camb)* 2019;1–8.

Harbuz R, Zouari R, Dieterich K, Nikas Y, Lunardi J, Hennebicq S, Ray P-F. [Function of aurora kinase C (AURKC) in human reproduction]. *Gynecol Obstet Fertil* 2009;**37**:546–551.

Huang L, Tong X, Luo L, Zheng S, Jin R, Fu Y, Zhou G, Li D, Liu Y. Mutation analysis of the TUBB8 gene in nine infertile women with oocyte maturation arrest. *Reprod Biomed Online* 2017;**35**:305–310.

Huang L, Tong X, Wang F, Luo L, Jin R, Fu Y, Zhou G, Li D, Song G, Liu Y, *et al.* Novel mutations in PATL2 cause female infertility with oocyte germinal vesicle arrest. *Hum Reprod* 2018;**33**:1183–1190.

Li L, Feng F, Wang Y, Guo J, Yue W. Mutational effect of human CFAP43 splice-site variant causing multiple morphological abnormalities of the sperm flagella. *Andrologia* 2020;**52**:e13575.

Liu C, He X, Liu W, Yang S, Wang L, Li W, Wu H, Tang S, Ni X, Wang J, *et al.* Bi-allelic Mutations in TTC29 Cause Male Subfertility with Asthenoteratospermia in Humans and Mice. *Am J Hum Genet* 2019;**105**:1168–1181.

Liu Z, Zhu L, Wang J, Luo G, Xi Q, Zhou X, Li Z, Yang X, Duan J, Jin L, *et al.* Novel homozygous mutations in PATL2 lead to female infertility with oocyte maturation arrest. *J Assist Reprod Genet* 2020;**37**:841–847.

Liu H, Wei X, Sha Y, et al. Whole-exome sequencing in patients with premature ovarian insufficiency: early detection and early intervention. *J Ovarian Res*. 2020;13(1):114. Published 2020 Sep 22.

Maddirevula S, Coskun S, Alhassan S, Elnour A, Alsaif HS, Ibrahim N, Abdulwahab F, Arold ST, Alkuraya FS. Female Infertility Caused by Mutations in the Oocyte-Specific Translational Repressor PATL2. *Am J Hum Genet* 2017;**101**:603–608.

Ortega V, Oyanedel J, Fleck-Lavergne D, Horta F, Mercado-Campero A, Palma-Ceppi C. Macrozoospermia associated with mutations of AURKC gene: First case report in Latin America and literature review. *Rev Int Androl* 2019;

Pu D, Wang C, Cao J, Shen Y, Jiang H, Liu J, Wu BL, Zhang W, Wu J. Association analysis between HFM1 variation and primary ovarian insufficiency in Chinese women. *Clin Genet* 2016;**89**:597–602.

Rocca MS, Msaki A, Ghezzi M, Cosci I, Pilichou K, Celeghin R, Foresta C, Ferlin A. Development of a novel next-generation sequencing panel for diagnosis of quantitative spermatogenic impairment. *J Assist Reprod Genet* 2020;**37**:753–762.

Sha Y, Yang X, Mei L, Ji Z, Wang X, Ding L, Li P, Yang S. DNAH1 gene mutations and their potential association with dysplasia of the sperm fibrous sheath and infertility in the Han Chinese population. *Fertil Steril* 2017;**107**:1312-1318.e2.

Sha Y-W, Wang X, Su Z-Y, Mei L-B, Ji Z-Y, Bao H, Li P. Patients with multiple morphological abnormalities of the sperm flagella harbouring CFAP44 or CFAP43 mutations have a good pregnancy outcome following intracytoplasmic sperm injection. *Andrologia* 2019;**51**:e13151.

Takasaki N, Tachibana K, Ogasawara S, Matsuzaki H, Hagiuda J, Ishikawa H, Mochida K, Inoue K, Ogonuki N, Ogura A, *et al.* A heterozygous mutation of GALNTL5 affects male infertility with impairment of sperm motility. *Proc Natl Acad Sci USA* 2014;**111**:1120–1125.

Tang S, Wang X, Li W, Yang X, Li Z, Liu W, Li C, Zhu Z, Wang L, Wang J, *et al.* Biallelic Mutations in CFAP43 and CFAP44 Cause Male Infertility with Multiple Morphological Abnormalities of the Sperm Flagella. *Am J Hum Genet* 2017;**100**:854–864.

Tu C, Nie H, Meng L, Yuan S, He W, Luo A, Li H, Li W, Du J, Lu G, *et al.* Identification of DNAH6 mutations in infertile men with multiple morphological abnormalities of the sperm flagella. *Sci Rep* 2019;**9**:15864.

Wang A-C, Zhang Y-S, Wang B-S, Zhao X-Y, Wu F-X, Zhai X-H, Sun J-X, Mei S-Y. Mutation analysis of the TUBB8 gene in primary infertile women with arrest in oocyte maturation. *Gynecol Endocrinol* 2018;**34**:900–904.

Wang X, Jin H, Han F, Cui Y, Chen J, Yang C, Zhu P, Wang W, Jiao G, Wang W, *et al.* Homozygous DNAH1 frameshift mutation causes multiple morphological anomalies of the sperm flagella in Chinese. *Clin Genet* 2017;**91**:313–321.

Wang J, Zhang W, Jiang H, Wu B-L, Primary Ovarian Insufficiency Collaboration. Mutations in HFM1 in recessive primary ovarian insufficiency. *N Engl J Med* 2014;**370**:972–974.

Wu L, Chen H, Li D, Song D, Chen B, Yan Z, Lyu Q, Wang L, Kuang Y, Li B, *et al.* Novel mutations in PATL2: expanding the mutational spectrum and corresponding phenotypic variability associated with female infertility. *Journal of human genetics* [Internet] 2019;**64**:. J Hum Genet.

Xiang J, Wang W, Qian C, Xue J, Wang T, Li H, Li H. Human oocyte maturation arrest caused by a novel missense mutation in TUBB8. *J Int Med Res* 2018;**46**:3759–3764.

Xing Q, Wang R, Chen B, et al. Rare homozygous mutation in TUBB8 associated with oocyte maturation defect-2 in a consanguineous mating family. *J Ovarian Res*. 2020;13(1):42.

Yang X, Zhu D, Zhang H, Jiang Y, Hu X, Geng D, Wang R, Liu R. Associations between DNAH1 gene polymorphisms and male infertility: A retrospective study. *Medicine (Baltimore)* 2018;**97**:e13493.

Yang P, Yin C, Li M, Ma S, Cao Y, Zhang C, Chen T, Zhao H. Mutation analysis of tubulin beta 8 class VIII in infertile females with oocyte or embryonic defects. Clin Genet. 2021 Jan;99(1):208-214.

Yanus GA, Akhapkina TA, Whitehead AJ, Bizin IV, Iyevleva AG, Kuligina ESh, Aleksakhina SN, Anisimova MO, Holmatov MM, Romanko AA, *et al.* Exome-based search for recurrent disease-causing alleles in Russian population. *European Journal of Medical Genetics* 2019;**62**:103656.

Yatsenko AN, Roy A, Chen R, Ma L, Murthy LJ, Yan W, Lamb DJ, Matzuk MM. Non-invasive genetic diagnosis of male infertility using spermatozoal RNA: KLHL10 mutations in oligozoospermic patients impair homodimerization. *Hum Mol Genet* 2006;**15**:3411–3419.

Yuan P, Zheng L, Liang H, Li Y, Zhao H, Li R, Lai L, Zhang Q, Wang W. A novel mutation in the TUBB8 gene is associated with complete cleavage failure in fertilized eggs. *J Assist Reprod Genet* 2018;**35**:1349–1356.

Zhao L, Guan Y, Wang W, et al. Identification novel mutations in TUBB8 in female infertility and a novel phenotype of large polar body in oocytes with TUBB8 mutations [published online ahead of print, 2020 Jun 10]. *J Assist Reprod Genet*. 2020. doi:10.1007/s10815-020-01830-6

Zhe J, Chen S, Chen X, Liu Y, Li Y, Zhou X, Zhang J. A novel heterozygous splice-altering mutation in HFM1 may be a cause of premature ovarian insufficiency. *J Ovarian Res* 2019;**12**:61.
